# Thrombopoietin mimetic stimulates bone marrow vascular and stromal niches to mitigate acute radiation syndrome

**DOI:** 10.1186/s13287-024-03734-z

**Published:** 2024-04-29

**Authors:** Justin Vercellino, Beata Małachowska, Shilpa Kulkarni, Brett I. Bell, Shahin Shajahan, Kosaku Shinoda, Gary Eichenbaum, Amit K. Verma, Sanchita P. Ghosh, Weng-Lang Yang, Paul S. Frenette, Chandan Guha

**Affiliations:** 1 Department of Radiation Oncology, Albert Einstein College of Medicine, 1300 Morris Park Avenue, Bronx, NY 10461 USA; 2 Department of Pathology, Albert Einstein College of Medicine, Bronx, NY USA; 3 Department of Molecular Pharmacology, Albert Einstein College of Medicine, Bronx, NY USA; 4 Johnson & Johnson, Office of the Chief Medical Officer, New Brunswick, NJ USA; 5 Department of Developmental and Molecular Biology, Albert Einstein College of Medicine, Bronx, NY USA; 6 Armed Forces Radiobiology Research Institute, Uniformed Services University of the Health Sciences, Bethesda, MD USA; 7 Department of Cell Biology, Albert Einstein College of Medicine, Bronx, NY USA; 8 Present Address: National Institute of Allergy and Infectious Diseases, National Institutes of Health, Rockville, MD USA; 9 Present Address: Bioconvergent Health, LLC, Purchase, NY USA

**Keywords:** Hematopoietic acute radiation syndrome, Total body irradiation, Bone marrow, Mesenchymal stromal cells, Endothelial cells, Thrombopoietin mimetic

## Abstract

**Background:**

Acute radiation syndrome (ARS) manifests after exposure to high doses of radiation in the instances of radiologic accidents or incidents. Facilitating regeneration of the bone marrow (BM), namely the hematopoietic stem and progenitor cells (HSPCs), is key in mitigating ARS and multi-organ failure. JNJ-26366821, a PEGylated thrombopoietin mimetic (TPOm) peptide, has been shown as an effective medical countermeasure (MCM) to treat hematopoietic-ARS (H-ARS) in mice. However, the activity of TPOm on regulating BM vascular and stromal niches to support HSPC regeneration has yet to be elucidated.

**Methods:**

C57BL/6J mice (9–14 weeks old) received sublethal or lethal total body irradiation (TBI), a model for H-ARS, by ^137^Cs or X-rays. At 24 h post-irradiation, mice were subcutaneously injected with a single dose of TPOm (0.3 mg/kg or 1.0 mg/kg) or PBS (vehicle). At homeostasis and on days 4, 7, 10, 14, 18, and 21 post-TBI with and without TPOm treatment, BM was harvested for histology, BM flow cytometry of HSPCs, endothelial (EC) and mesenchymal stromal cells (MSC), and whole-mount confocal microscopy. For survival, irradiated mice were monitored and weighed for 30 days. Lastly, BM triple negative cells (TNC; CD45^−^, TER-119^−^, CD31^−^) were sorted for single-cell RNA-sequencing to examine transcriptomics after TBI with or without TPOm treatment.

**Results:**

At homeostasis, TPOm expanded the number of circulating platelets and HSPCs, ECs, and MSCs in the BM. Following sublethal TBI, TPOm improved BM architecture and promoted recovery of HSPCs, ECs, and MSCs. Furthermore, TPOm elevated VEGF-C levels in normal and irradiated mice. Following lethal irradiation, mice improved body weight recovery and 30-day survival when treated with TPOm after ^137^Cs and X-ray exposure. Additionally, TPOm reduced vascular dilation and permeability. Finally, single-cell RNA-seq analysis indicated that TPOm increased the expression of collagens in MSCs to enhance their interaction with other progenitors in BM and upregulated the regeneration pathway in MSCs.

**Conclusions:**

TPOm interacts with BM vascular and stromal niches to locally support hematopoietic reconstitution and systemically improve survival in mice after TBI. Therefore, this work warrants the development of TPOm as a potent radiation MCM for the treatment of ARS.

**Supplementary Information:**

The online version contains supplementary material available at 10.1186/s13287-024-03734-z.

## Background

Exposure to ionizing radiation, whether from accidental incidents or as a preparative regimen for allogenic stem cell transplantation to treat leukemias, results in profound bone marrow (BM) injury. Total body irradiation (TBI) can affect various organ systems, with the hematopoietic system being the most radiosensitive [1, 2]. Preserving and reconstituting hematopoietic stem and progenitor cells (HSPCs) in the BM is crucial for mitigating mortality from hematopoietic acute radiation syndrome (H-ARS), typically occurring after high-dose TBI over a short period of time [3, 4]. HSPCs comprise all blood and immune cells which support the vital function of eliminating infection among many others [5–8]. A comprehensive understanding of the mechanisms and biochemical pathways governing HSPC regeneration is essential for developing life-saving medical countermeasures (MCMs) against H-ARS and mitigating radiation injuries in clinical applications where radiation is used.

The BM microenvironment consists hematopoietic, vascular, and stromal niches which support and nurture each other [9–11]. HSPCs can be broadly classified into myeloid progenitor cells (MPC) and lineage^−^, Sca-1^+^, c-kit^+^ (LSK) cells. The LSK population consists of short-term ‘cycling’ HSCs (ST-HSCs) and long-term ‘quiescent’ HSCs (LT-HSCs) [12–14]. For non-hematopoietic cells, the vascular niche is comprised of endothelial progenitor cells (EPCs) and endothelial cells (ECs) that release canonical niche factors such as stem cell factor (SCF), CXCL12, and angiopoietin-1 to support HSPCs [14–21]. Additionally, the BM vascular niche can be divided into the sinusoidal niche, harboring both quiescent and proliferative HSCs and serving as the main site of BM entry and egress; and the arteriolar niche, supporting quiescent HSCs around small arterioles near the endosteal region of the BM [5, 12, 22]. Furthermore, perivascular mesenchymal stromal cells (MSCs) belonging to the stromal niche represent a small, but essential part of the CD45^−^TER-119^−^CD31^−^ (triple negative cells; TNC) fraction in the BM [23]. MSCs secrete growth factors and chemokines such as vascular endothelial growth factors (VEGFs), CXCL12, and SCF that support both BM ECs and HSPCs [24, 25]. High doses of ionizing radiation are known for inducing vascular injury; however, in-depth mechanistic studies focused on BM vascular niche injury have been limited, even more so for the stromal niche. Thus, therapeutic strategies aimed at mitigating BM vascular and stromal damage are currently lacking.

Thrombopoietin (TPO), a glycoprotein hormone and pleiotropic growth factor, binds to its receptor, c-MPL, expressed on megakaryocytes, platelets, and HSPCs [26–28]. TPO’s primary biological function is to stimulate the generation of platelets [29, 30]. Clinical use of recombinant human TPO (rhTPO) for treating immune thrombocytopenia was discontinued due to subjects developing endogenous neutralizing antibodies, leading to immune-mediated thrombocytopenia [31]. Consequently, a class of drugs with low immunogenicity, referred to as TPO mimetics, were developed to stimulate c-MPL signaling [32]. JNJ-26366821, a TPO mimetic peptide (hereafter referred to as TPOm), is comprised of 29-amino acids conjugated to polyethylene glycol moieties with no sequence homology to endogenous TPO [33, 34]. Both rhTPO and other TPO mimetics, including JNJ-26366821, have demonstrated efficacy in mitigating H-ARS in murine and non-human primate models [35–39]. Currently, the impact of TPO on the BM vascular and stromal niches for HSPC regeneration after irradiation has remained largely unexplored.

In this study, we hypothesized that TPOm will promote regeneration of endothelial and stromal cells in the BM to facilitate hematopoietic recovery after TBI. As such, we investigated the impact of TPOm on megakaryocytes, hematopoietic, endothelial, and stromal cell populations in the BM of healthy and TBI mice using histopathology and flow cytometry. We evaluated the efficacy of TPOm given subcutaneously 24 h post-irradiation in enhancing the survival of TBI mice exposed to varying doses of ^137^Cs and X-rays. In addition, we applied in situ whole-mount confocal microscopy to examine the effect of TPOm on the architecture of the arteriolar and sinusoidal vessels with high spatio-temporal resolution in the BM. We also measured the levels of VEGF-A and C in the BM and serum and employed IVIS (In Vivo Imaging System) to evaluate TPOm’s effect on vascular permeability post-TBI. Lastly, we analyzed the effects and interactions of MSCs in the CD45^−^TER-119^−^CD31^−^ (triple negative cells; TNC) fraction with other hematopoietic TNCs using single-cell RNA-sequencing (scRNA-seq). This study is the first to reveal a novel activity of TPOm in fostering the interactions between MSCs and other progenitors within the BM, thereby sustaining BM vasculature and niche homeostasis, leading to HSPC regeneration and mitigation of H-ARS.

## Results

### TPOm expands hematopoietic, endothelial, and stromal cells in murine bone marrow at homeostasis

**Fig. 1:**
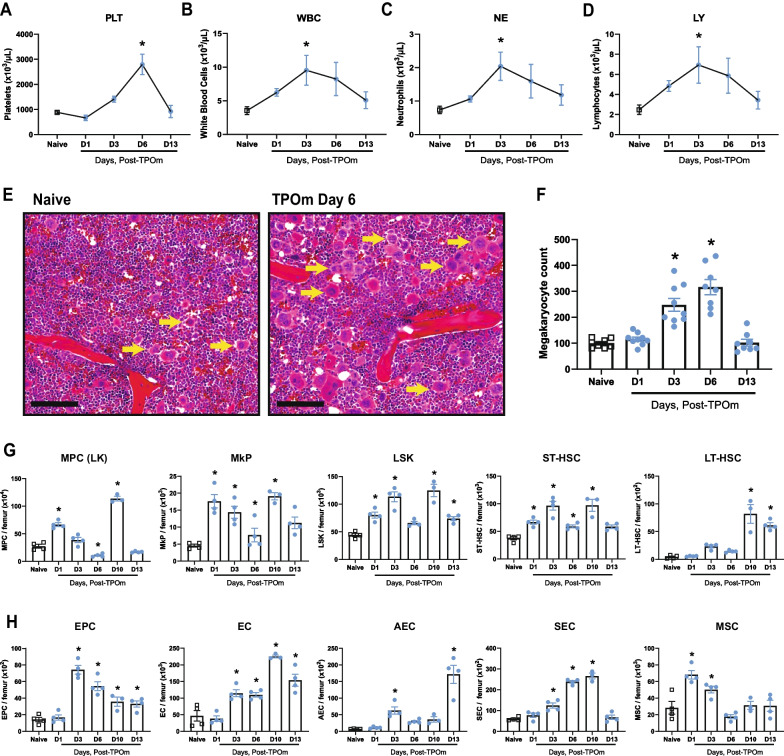
TPOm expands megakaryocytes, hematopoietic stem and progenitor, endothelial, and stromal cells in murine bone marrow at homeostasis. **A**–**D** Complete blood count of peripheral blood for **A** platelets, **B** white blood cells, **C** neutrophils, and **D** lymphocytes. **E** Representative H&E images of sternal bone marrow from naïve and mice treated with TPOm on day 6 after injection. Yellow arrows, megakaryocytes. Scale bar is 100 μm. (**F**) Count of megakaryocytes in the sternal bone marrow of naive and TPOm-treated mice at the indicated day post-injection (n = 3/group). **G** The number of MPC, MkP, LSK, ST-, and LT-HSC per femur of naive and TPOm-treated mice (n = 3–4/group) over time. **H** The number of EC, EPC, AEC, SEC, and MSC per femur of naive and TPOm-treated mice (n = 3–4/group) over time. Data are expressed as mean ± SEM. *p < 0.05 *vs.* naïve assessed by one-way ANOVA with post hoc Dunnett test for multiple comparisons

To confirm the biological activity of TPOm through the TPO/c-MPL pathway, we initially measured platelets in the peripheral blood of normal C57BL/6J mice on days 1, 3, 6, and 13 following a single subcutaneous (*sc*) injection of TPOm dosed at 0.3 mg/kg. As expected, platelets (PLT) gradually increased over time, reaching a 3.2-fold peak compared to naïve 6 days after TPOm treatment (Fig. 1A). Furthermore, the numbers of white blood cells (WBC), neutrophils (NE), and lymphocytes (LY) increased ~ 2.7-fold 3 days after TPOm treatment compared to naïve (Fig. 1B–D). Considering platelets arise from megakaryocytes, we then examined them in the BM after TPOm treatment. Histological analysis of sternal marrow with H&E revealed a significant 2.6- and 3.2-fold increase in megakaryocytes on days 3 and 6 post-TPOm treatment, respectively, compared to naïve (Fig. 1E, F). Morphologically in the TPOm-treated mice, the megakaryocytes are mostly mature, polylobated with abundant cytoplasm. A few young, smaller mononuclear megakaryocytes can also be visualized with rare mitoses. The number of megakaryocytes in the TPOm-treated mice returned to baseline by day 13 (Fig. 1F).

As the number of BM megakaryocytes increased following TPOm treatment, we subsequently investigated the expansion of HSPCs in BM. The HSPC populations were assessed by flow cytometry on days 1, 3, 6, and 13 post-TPOm treatment with the gating strategy depicted in Additional file 1: Figure S1A. MPCs (lineage^−^ c-kit^+^), exhibited a significant increase on day 1, followed by a significant depletion on day 6 after TPOm treatment relative to naïve mice (Fig. 1G and Additional file 1: Figure S1G). Despite this decrease, there was a significant, transient expansion of MPCs on day 10, returning to baseline by day 13 after TPOm treatment (Fig. 1G and Additional file 1: Figure S1B). The LSK, megakaryocyte progenitors (MkPs), and ST-HSCs (LSK, CD34^+^) exhibited a similar trend to MPCs after TPOm treatment (Fig. 1G and Additional file 1: Figure S1B). Lastly, LT-HSCs (LSK, CD34^−^CD48^−^CD150^+^) only showed a significant increase by days 10 and 13 post-TPOm treatment (Fig. 1G and Additional file 1: Figure S1B).

The BM vascular and stromal niches also plays a crucial role in maintaining and supporting HSPCs at homeostasis. Thus, we investigated the impact of TPOm on EPCs, ECs, and MSCs using flow cytometry on days 1, 3, 6, and 13 post-TPOm treatment with the gating strategy outlined in Additional file 1: Figure S1C. Post-TPOm treatment, EPCs (CD45^−^, TER-119^−^, CD31^+^, CD34^+^, VEGFR2^+^) peaked with a 5.2-fold increase on day 3, while ECs (CD45^−^, TER-119^−^, CD31^+^) peaked with a 4.8-fold increase on day 10 (Fig. 1H and Additional file 1: Figure S1D). Analysis of vascular subsets within ECs, including arteriolar endothelial cells (AECs; CD45^−^, TER-119^−^, CD31^+^, CD62P^−/low^, Sca-1^+^) and sinusoidal endothelial cells (SECs; CD45^−^, TER-119^−^, CD31^+^, CD62P^+^, Sca-1^−/low^), revealed a significant increase by day 3 post-TPOm injection, with SECs peaking on day 10 (Fig. 1H and Additional file 1: Figure S1D). Further, MSCs (CD45^−^, TER-119^−^, CD31^−^, CD51^+^, CD140α^+^) were acutely expanded on days 1 and 3 after TPOm treatment, returning to baseline by day 6 (Fig. 1H). These findings collectively demonstrate that TPOm effectively increases megakaryocytes and HSPCs in vivo. Remarkably, endothelial and stromal cells in the BM of healthy mice also expanded after TPOm treatment suggesting a broader effect of TPOm on the niche.

### TPOm preserves murine bone marrow architecture and facilitates recovery of hematopoietic, endothelial, and stromal cells after sublethal irradiation

**Fig. 2:**
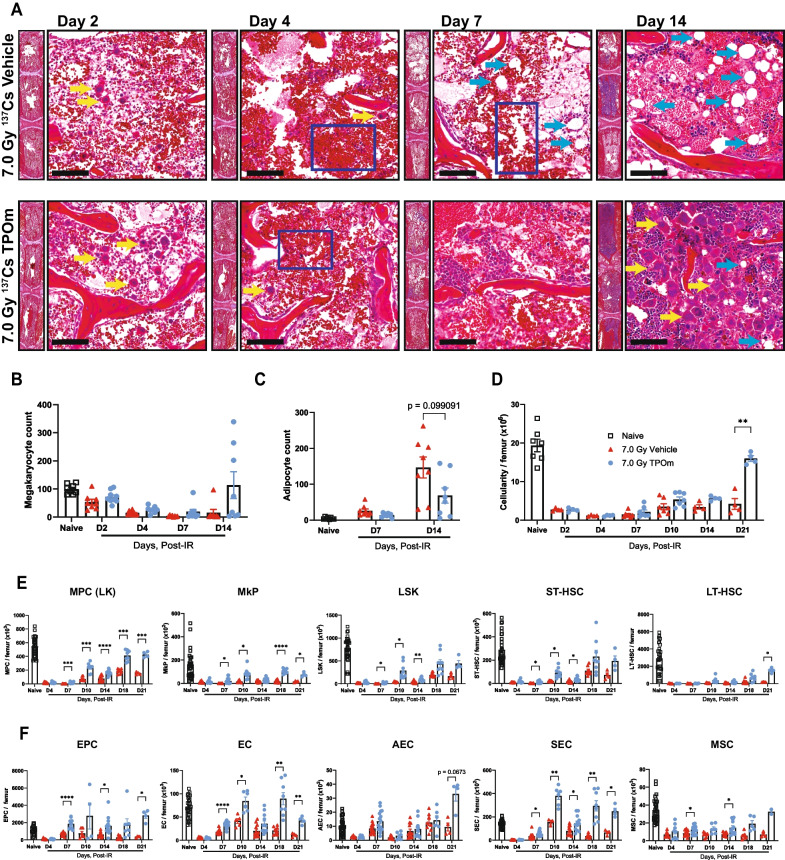
TPOm promotes recovery of hematopoietic stem and progenitor, endothelial, and stromal cells in murine bone marrow following 7.0 Gy sublethal total body irradiation. **A** Representative H&E images of sternal bone marrow from vehicle and TPOm-treated mice on days 2, 4, 7, and 14 after irradiation. Yellow arrow, megakaryocytes; light blue arrow, adipocytes; dark blue box, hemorrhaging. Scale bar is 100 μm. **B** The number of megakaryocytes in the sternal bone marrow of naïve, vehicle, and TPOm-treated mice over time (n = 3/group). **C** The number of adipocytes using MarrowQuant through QuPath in the sternal bone marrow of naïve, vehicle, and TPOm-treated mice over time (n = 3/group). **D** Live cell count of femoral bone marrow of naïve, vehicle, and TPOm-treated mice over time (n = 4/group). **E** The number of MPC, MkP, LSK, ST-, and LT-HSC per femur of naïve, vehicle, and TPOm-treated mice (n = 4–29/group) over time. **F** The number of EC, EPC, AEC, SEC, and MSC per femur of naïve, vehicle, and TPOm-treated mice (n = 4–29/group) over time. Data are expressed as mean ± SEM. *p < 0.05, **p < 0.01, ***p < 0.001, ****p < 0.0001 vehicle *vs*. TPOm-treated assessed by unpaired Student’s *t*-test with post hoc Holm-Sidak method for multiple comparisons. Outliers were determined using ROUT with a Q = 0.2%

Having established TPOm’s capacity to expand HSPC, EC, and MSC populations in the BM of healthy mice, we subsequently investigated its potential to restore the BM of mice subjected to sublethal TBI. Following TBI with ^137^Cs dosed at 7 Gy, mice received a single *sc* dose of TPOm at 0.3 mg/kg 24 h post-irradiation and assessed on days 2, 4, 7, and 14 post-TBI. First, the integrity of BM in irradiated mice was examined over time by H&E histology. On day 2 post-TBI, no gross differences were observed between vehicle- and TPOm-treated mice (Fig. 2A). On day 4 post-TBI, the size and extent of hemorrhage in the vehicle-treated mice were greater than the TPOm-treated mice (Fig. 2A). On day 7 post-TBI, early hematopoietic regeneration was evident near the endosteum in TPOm-treated mice, while not yet found in the vehicle-treated mice (Fig. 2A). Lastly, on day 14 after TBI, expansion of megakaryocytes was observed in the sternebrae of TPOm-treated mice, but not as prominently in the vehicle (Fig. 2A, B). Morphologically, the megakaryocytes resemble those present in the non-irradiated mice after TPOm treatment (Fig. 1E). Moreover, an increase in adipocytes was noted in the irradiated marrow on day 14 in both groups (Fig. 2A). Quantifying adipocytes using MarrowQuant [40] identified more adipocytes in the vehicle-treated BM relative to the TPOm-treated BM on day 14 (Fig. 2C). Assessment of cellularity, determined by counting live cells per femur, indicated a significant recovery on day 21 after TBI in the TPOm-treated mice, showing a 3.8-fold increase relative to vehicle-treated (Fig. 2D).

To precisely evaluate the impact of irradiation with and without TPOm on HSPC, EC, and MSC populations in the BM, we conducted flow cytometry. In TPOm-treated mice, the frequency of MPCs significantly increased by day 10 after irradiation (Additional file 1: Figure S2A), while the absolute count surpassed that of the vehicle-treated mice from day 7 through day 21 after TBI (Fig. 2E). Similarly, the absolute count of MkPs in TPOm-treated mice significantly exceeded that in vehicle-treated mice from day 7 to day 21 after TBI, excluding day 14 (Fig. 2E). For the LSK population, a significant increase in frequency was observed on days 7 and 10 in the TPOm-treated mice (Additional file 1: Figure S2A), while their absolute counts remained elevated through day 14 relative to vehicle-treated mice (Fig. 2E). ST-HSCs exhibited a similar trend to LSK cells (Fig. 2E). Conversely, for the rare LT-HSCs, a significant increase in both frequency and absolute count was only observed on day 21 after TBI in TPOm-treated mice compared to vehicle-treated mice (Fig. 2E and Additional file 1: Figure S2A).

In the BM vascular and stromal niches, EPCs in TPOm-treated mice exhibited a significant increase in both frequency and absolute count on day 7, compared to vehicle-treated mice (Fig. 2F and Additional file 1: Figure S2B). Total BM ECs showed a significant increase in frequency only on day 7 in TPOm-treated mice compared to the vehicle-treated mice (Additional file 1: Figure S2B). Moreover, the absolute count of total ECs in TPOm-treated mice was significantly higher than the vehicle-treated mice from days 7 through 21, with a trend for higher counts on day 14 that was not significant (Fig. 2F). For the subsets of ECs, the absolute count of AECs showed no statistical difference between vehicle- and TPOm-treated mice, while the absolute count of SECs significantly increased in TPOm-treated mice from day 7 through day 21 (Fig. 2F). Finally, the absolute count of MSCs also significantly increased on days 7 and 14 in TPOm-treated mice compared to the vehicle-treated mice after TBI (Fig. 2F). These results emphasize TPOm’s role in stimulating repopulation and regeneration of HSPCs, ECs, and MSCs in the BM, thereby contributing to the preservation of the BM architecture in mice after sublethal irradiation.

### TPOm increases survival of mice exposed to lethal irradiation

**Fig. 3:**
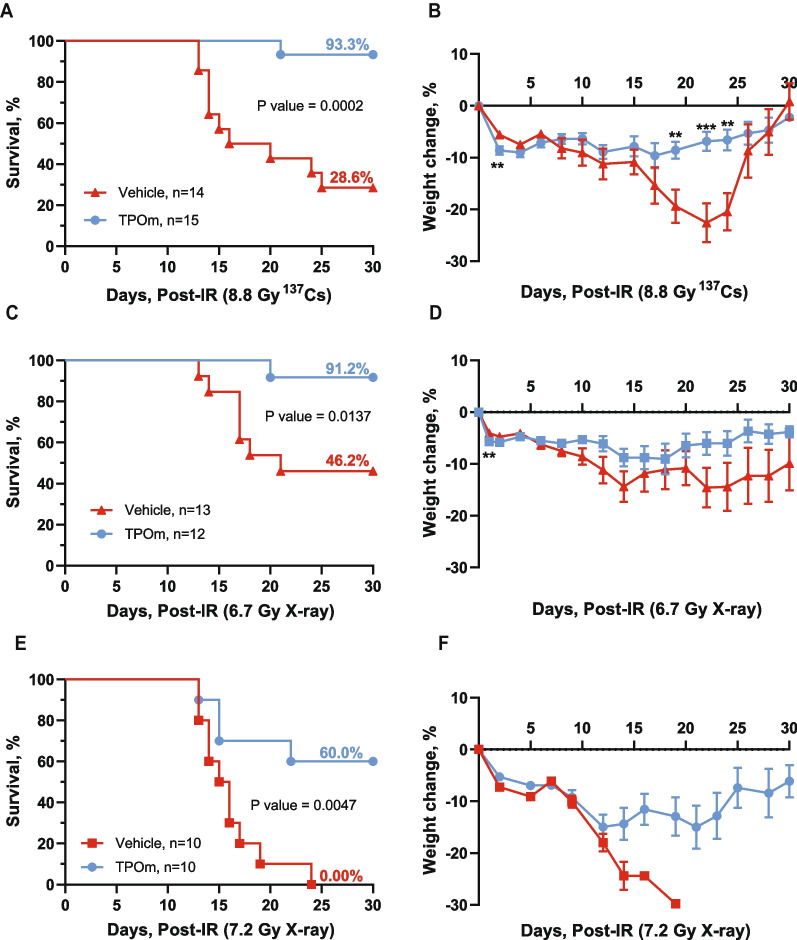
TPOm significantly increases survival of mice after lethal total body irradiation. Kaplan–Meier survival curve of vehicle and TPOm treated male mice for 30 days after **A** 8.8 Gy ^137^Cs, **C** 6.7 Gy X-ray, and **E** 7.2 Gy X-ray TBI. The percentage of body weight change over 30 days after **B** 8.8 Gy ^137^Cs, **D** 6.7 Gy X-ray, and **F** 7.2 Gy X-ray TBI. For survival, the Log-rank (Mantel-Cox) test was used for curve comparison. For the percent weight change data are expressed as mean ± SEM. *p < 0.05, **p < 0.01, ***p < 0.001, vehicle *vs.* TPOm-treated by unpaired Student’s *t*-test with post hoc Holm-Sidak method for multiple comparisons

As TPOm demonstrates the capability to mitigate BM damage in mice following sublethal irradiation, we explored its potential as a radiation MCM for H-ARS. The primary endpoint for evaluating the efficacy of a radiation MCM candidate in animal models is by assessing its impact on the 30-day survival post-lethal TBI. To evaluate the efficacy of TPOm, C57BL/6J mice were irradiated with lethal dose of TBI at 8.8 Gy from a ^137^Cs source. This radiation dose was selected because 8.8 Gy TBI is the lethal dose for 70% (LD_70_) of mice within 30 days post-TBI. Twenty-four hours post-TBI, mice received a single *sc* dose of TPOm at 0.3 mg/kg or vehicle. As illustrated in Fig. 3A, TPOm treatment significantly increased the survival from 28.6% in vehicle-treated mice to 93.3% in the TPOm-treated mice. Moreover, TPOm treatment significantly prevented body weight loss compared to the vehicle-treated mice (Fig. 3B).

We further assessed the efficacy of TPOm in enhancing survival in mice exposed to X-ray TBI. The dose of LD_100_ using orthovoltage X-rays is lower than ^137^Cs, as established in our previous publication [41]. Notably, a significant increase in survival was observed in male mice exposed to 6.7 and 7.2 Gy TBI (LD_50_ and LD_100_, respectively) with TPOm treatment compared to the vehicle-treated mice (Fig. 3C, E). Additionally, the male surviving mice maintained their body weight after irradiation (Fig. 3D, F). In female mice, TPOm improved the survival from 10 to 50% post-7.2 Gy TBI, although it was not statistically significant (Additional file 1: Figure S3A, *p* = 0.0681). Moreover, there was no significant difference in the percentage of body weight loss between vehicle- and TPOm-treated TBI female mice (Additional file 1: Figure S3B).

Upon closer examination of BM damage after 8.8 Gy (^137^Cs) TBI by H&E histology, we noted a substantial reduction in BM cellularity in both vehicle- and TPOm-treated mice on day 4 (Additional file 1: Figure S3C). On day 14 after TBI, sinusoidal dilatation was evident in both groups. However, TPOm-treated mice exhibited more defined, intact vessels, known as BM angiectasis. In contrast, vehicle-treated mice displayed extensive hemorrhaging of the vessels with erythrocytes present in the parenchyma, indicating compromised BM sinusoids (Additional file 1: Figure S3C). Overall, these data demonstrate that TPOm can serve as a potent radiation MCM by increasing the survival of mice exposed to a lethal dose of radiation from various sources.

### TPOm accelerates and promotes restoration of the vascular niche in mice after lethal irradiation

**Fig. 4:**
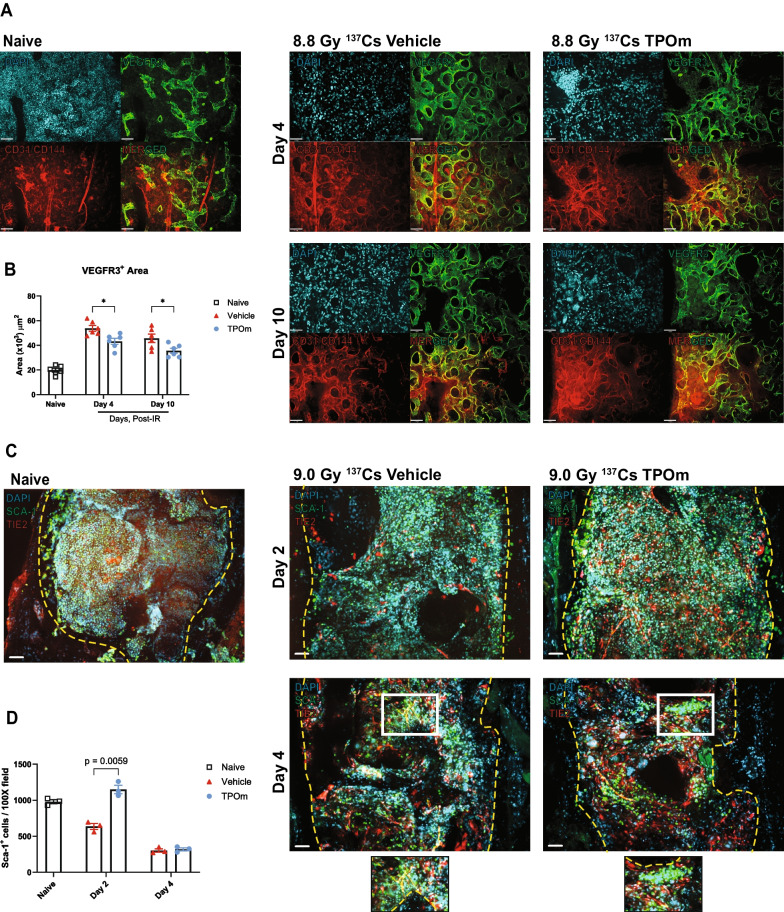

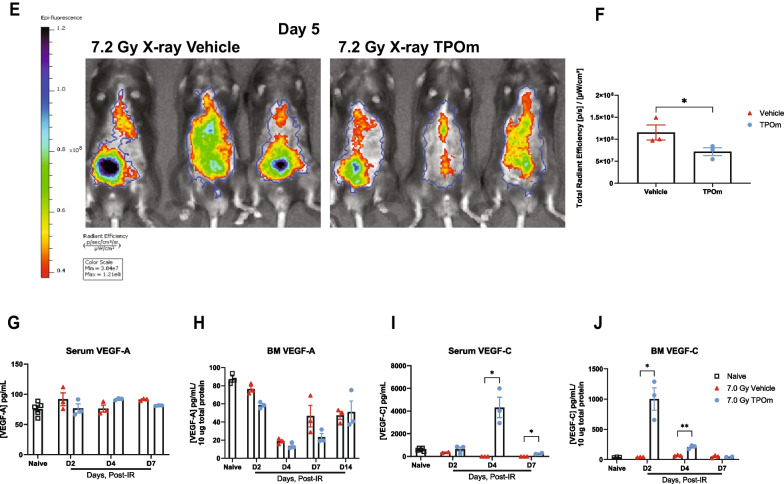
TPOm systemically reduces bone marrow vascular dilatation and vascular leakage and promotes production of VEGF-A and VEGF-C in irradiated mice. **A** Representative immunofluorescent images of femurs stained with DAPI (blue), VEGFR3 (green), and CD31/CD144 (red) on days 4 and 10 after irradiation in vehicle and TPOm-treated mice. Non-irradiated mice were represented as naïve for reference. Scale bar is 10 μm. **B** VEGFR3^+^ vessel area in the bone marrow on days 4 and 10 after irradiation, quantitated by using Volocity software (n = 3/group). **C** Representative immunofluorescent images of sternum stained with DAPI (blue), Sca-1 (green), and TIE2 (red) on days 2 and 4 after irradiation in vehicle and TPOm-treated mice from 2 independent experiments. Non-irradiated mice were represented as naïve for reference. **D** Quantification of Sca-1^+^ cells per 100 × field in sternal bone marrow quantitated by using Volocity software (n = 3/group). **E** IVIS images of 7.2 Gy (X-rays) TBI mice imaged 5 days after irradiation with AngioSense750 EX i.v. injection performed on day 3 after irradiation. **F** Quantification of total radiant efficiency (n = 3/group). **G**, **H** ELISA of VEGF-A in (**G**) serum and in (**H**) BM after 7.0 Gy (^137^Cs) TBI. **I**, **J** ELISA of VEGF-C in (**I**) serum and in (**J**) BM after 7.0 Gy (^137^Cs) TBI. Data are expressed as mean ± SEM. *p < 0.05, **p < 0.01 vehicle *vs*. TPOm-treated assessed by unpaired Student’s *t*-test with post hoc Holm-Sidak method for multiple comparisons

Extensive vascular dilatation and an increase in vascular area in the diaphysis are inherent responses to BM stress and injury, particularly for the BM sinusoids [9]. Therefore, we investigated the sinusoidal niche in situ using whole-mount confocal microscopy of the femur. BM SECs were identified by vascular endothelial growth factor receptor 3 (VEGFR3) labeling, which is exclusively expressed on the BM sinusoids (Fig. 4A). Combined labeling of CD31 and CD144 was employed to examine the total BM vasculature, with DAPI used for nuclei identification in mice irradiated at 8.8 Gy (^137^Cs) TBI. The VEGFR3^+^-stained area in naïve mice was 19.8 ± 1.45 × 10^3^ µm^2^ (Fig. 4A, B). By day 4 after irradiation, this area increased to 53.7 ± 2.30 × 10^3^ µm^2^ in vehicle-treated mice, while TPOm significantly inhibited the dilation to 43.3 ± 2.39 × 10^3^ µm^2^ (Fig. 4A, B). Furthermore, by day 10, the VEGFR3^+^-stained area of vehicle-treated mice reached 45.7 ± 3.36 × 10^3^ µm^2^, while with TPOm, it was further reduced to 35.6 ± 2.08 × 10^3^ µm^2^ (Fig. 4A, B). These results demonstrate that TPOm enhances the restoration of BM sinusoids in irradiated animals.

To assess the effect of lethal TBI at 9.0 Gy (^137^Cs) on the arteriolar niche, we used angiopoietin-1 receptor, known as TIE2, as a marker to distinguish AECs, given their high expression of TIE2 [42]. BM arterioles also express Sca-1, typically a marker of for hematopoietic progenitors (Additional file 1: Figure S1A). On days 2 and 4 after TBI, arterioles, marked as Sca-1^+^ and TIE2^+^, were readily detected in both naive and irradiated mice and appeared unchanged (Fig. 4C). Moreover, on day 4, the Sca-1^+^ hematopoietic cells were found more clustered in the TPOm-treated mice compared to the vehicle, particularly around the arterioles (Fig. 4C). The number of Sca-1^+^ cells in the vehicle mice began to decrease on day 2 and further decreased on day 4, compared to the naïve (Fig. 4D). In contrast, the number of Sca-1^+^ cells in the TPOm-treated mice were well maintained, reaching a level similar to the naïve mice and significantly higher than the vehicle (637 ± 41 *vs.* 1150 ± 58 cells per field) on day 2, although it dropped to similar counts as the vehicle on day 4 (Fig. 4D). Despite not detecting noticeable changes in the BM arterioles after irradiation, there were increased numbers of Sca-1^+^ cells near the endosteum and arterioles in TPOm-treated mice.

To further analyze the impact of TPOm on vascular integrity and function, we utilized IVIS imaging to evaluate vascular permeability. Mice were exposed to 7.2 Gy TBI with X-rays, followed by TPOm treatment 24 h post-irradiation. On day 3 after irradiation, mice received an injection of a vascular dye (AngioSense 750EX) intravenously and were imaged 48 h later. The IVIS imaging showed that there was a greater amount of dye present in the tissues of the vehicle-treated mice compared to the TPOm-treated mice (Fig. 4E). After quantification of the region of interests (ROIs), the total radiant efficiency in the TPOm-treated group was significantly lower than the vehicle-treated group by 53.2% (Fig. 4F). These findings suggest that TPOm contributes to vascular integrity, as evidenced by the reduced leakage of vascular dye into surrounding tissues.

VEGFs play a crucial role in regulating ECs, influencing growth and repair processes [43]. Consequently, we investigated the effect of TPOm on the levels of VEGF-A and VEGF-C in the BM and serum. In healthy mice, following TPOm injection, VEGF-A levels significantly increased on day 3 in serum and on day 13 in the BM (Additional file 1: Figure S4A, B). Concurrently, VEGF-C levels significantly increased on days 1 and 3 in the BM and serum, respectively, after TPOm treatment (Additional file 1: Figure S4C, D). However, when mice were subjected to 7 Gy (^137^Cs) TBI, TPOm treatment did not elevate VEGF-A levels in either serum or BM, unlike healthy mice (Fig. 4G, H). Nevertheless, the levels of VEGF-C in the BM and serum of TPOm-treated mice were markedly increased on days 2 and 4, respectively, compared to the vehicle-treated and naïve mice (Fig. 4I, J). These results highlight that TPOm can selectively stimulate the release of VEGFs, distinctively VEGF-C, both systemically and locally, promoting the repair of vascular damage in the BM and potentially other organs after irradiation.

### TPOm elicits distinct changes in cellular heterogeneity and cell cycle dynamics of murine bone marrow cells post-irradiation evaluated by single-cell RNA-sequencing analysis

**Fig. 5:**
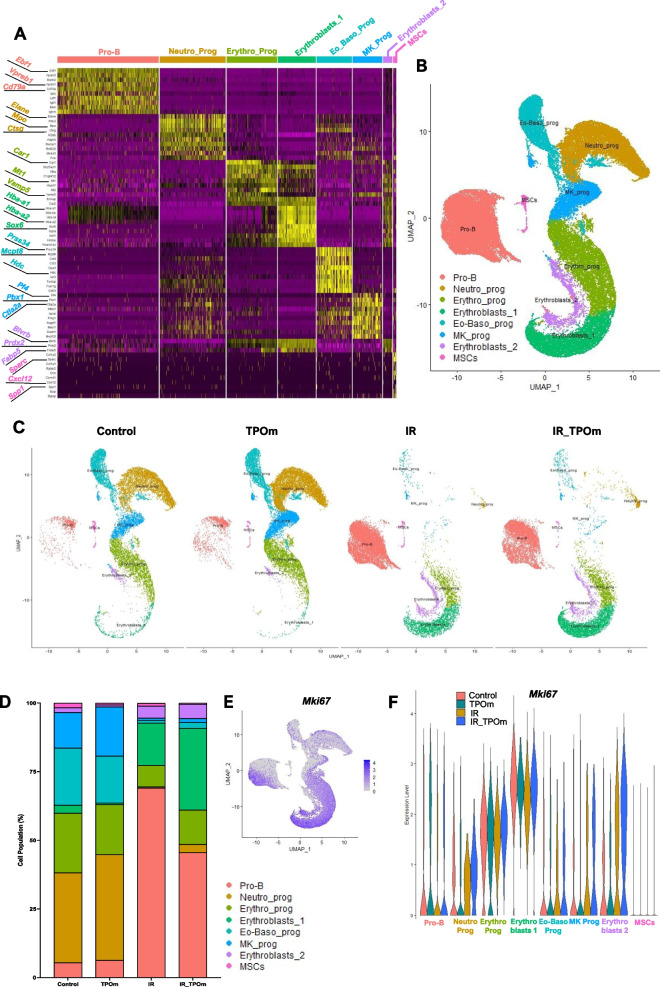

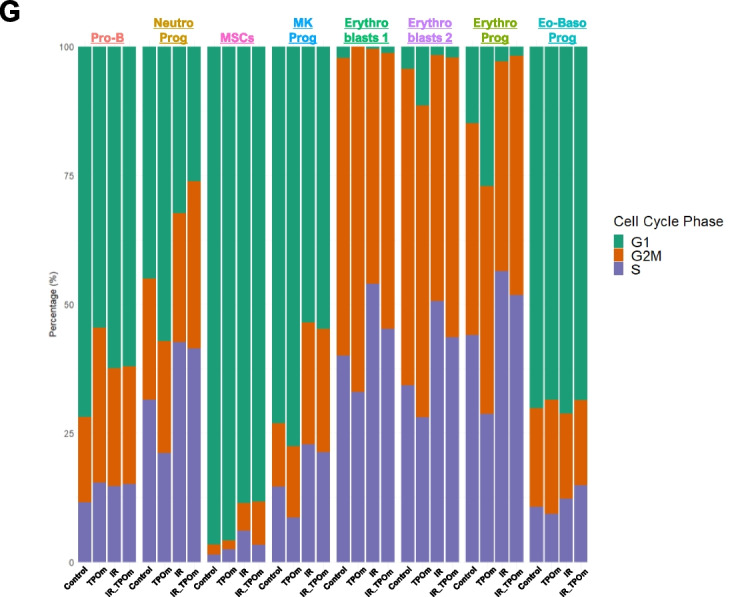
TPOm increases subpopulations of hematopoietic progenitors and *Mki67* expression in sorted bone marrow TNCs from mice after sublethal total body irradiation. Single-cell RNA-seq analysis of sorted CD45^−^, TER-119^−^, CD31^−^ (TNC) cells derived from BM of mice 10 days after 6 Gy (X-ray) TBI. **A** Heatmap of highly expressed genes used to identify different cell clusters. **B** Overall UMAP clustering of TNC cells, **C** individual UMAP clusters following each group: naïve (Control), TPOm alone, irradiated (IR), and irradiated plus TPOm-treated (IR_TPOm). **D** Each of the cluster’s distribution by percentage iterated by treatment condition. **E** Expression distribution of *Mki67* on the overall UMAP of TNC cells. **F** Violin plots of *Mki67* expression iterated by identified clusters per treatment group. **G** Cell cycle analysis of each identified cluster per treatment group

BM MSCs play a significant role in the regeneration of HSPCs and ECs, particularly after irradiation [24, 44]. Given the observed increase in MSCs after TPOm treatment (Figs. 1H and 2F), we further investigated the effect of TPOm on MSCs. We sorted BM from mice using the markers CD45^−^, TER-119^−^, and CD31^−^ [triple negative cells (TNC)] [23, 45] as this fraction is enriched for MSCs and conducted single-cell RNA-sequencing. Mice were divided into four groups: naïve, TPOm alone, 6 Gy TBI (X-rays), and 6 Gy TBI followed by TPOm treatment 24 h post-irradiation. BM was harvested on day 10 after irradiation. A heatmap of the top 10 enriched genes was generated for each cluster to identify the populations of TNCs (Fig. 5A). An overall UMAP was generated by combining clusters from all four groups (Fig. 5B) as well as individual UMAPs for each group (Fig. 5C). Notably, neutrophil progenitors (Neutro_prog), megakaryocyte progenitors (Mk_prog), and eosino-basophil progenitors (Eo-Baso_prog) were noticeably depleted after irradiation, while Pro-B cells and both clusters of erythroblasts were increased (Fig. 5C). Analyzing the percentage of each cluster per group revealed that TPOm alone increased the percentage of Neutro_prog and Mk_prog from 32.9% to 38.5% and 13% to 17.9%, respectively, compared to naïve (Fig. 5D). Following irradiation, most identified clusters, with the exception of Pro-B cells, were increased after TPOm treatment relative to the irradiation alone group (Fig. 5D).

To further pinpoint clusters demonstrating active proliferation, we analyzed the expression of the proliferation marker *Mki67*. The combined UMAP displayed elevated levels of *Mki67* in the erythroblasts and Pro-B cells (Fig. 5E). As anticipated, TPOm increased *Mki67* expression in Mk_prog cluster (Additional file 1: Figure S5A). Irradiation increased *Mki67* expression in Neutro_prog, Mk_prog, and erythroblast 2 clusters, an effect further amplified in the irradiation plus TPOm-treated group (Fig. 5F and Additional file 1: Figure S5A). Additionally, we assessed the cell cycle status of each cluster for all the individual groups (Additional file 1: Figure S5B). For comparison, the percentage of each cell cycle phase (G1, G2M, or S) in each cluster was plotted for all groups (Fig. 5G). In healthy mice, TPOm notably increased the percentage of Pro-B cells in the G2M phase, Neutro_prog in the G1 phase, and erythro- progenitors (Erythro_prog) in the G1 phase (Fig. 5G). Radiation increased the percentages of several clusters in S phase, while TPOm treatment slightly decreased the percentage of all the Erythro-clusters in the S phase (Fig. 5G). Particularly, MSCs exhibited a slight increase in the S phase after TPOm treatment in the irradiated groups; however, the percentage of MSCs in G2M was increased with TPOm treatment compared to irradiation alone (Fig. 5G). Collectively, these data highlight that TPOm regulates both the proliferation and cell cycle dynamics of erythroid, B lymphoid, and MSCs after irradiation.

### TPOm enhances the interaction of mesenchymal stromal cells with other hematopoietic progenitors in the mouse bone marrow after irradiation

**Fig. 6:**
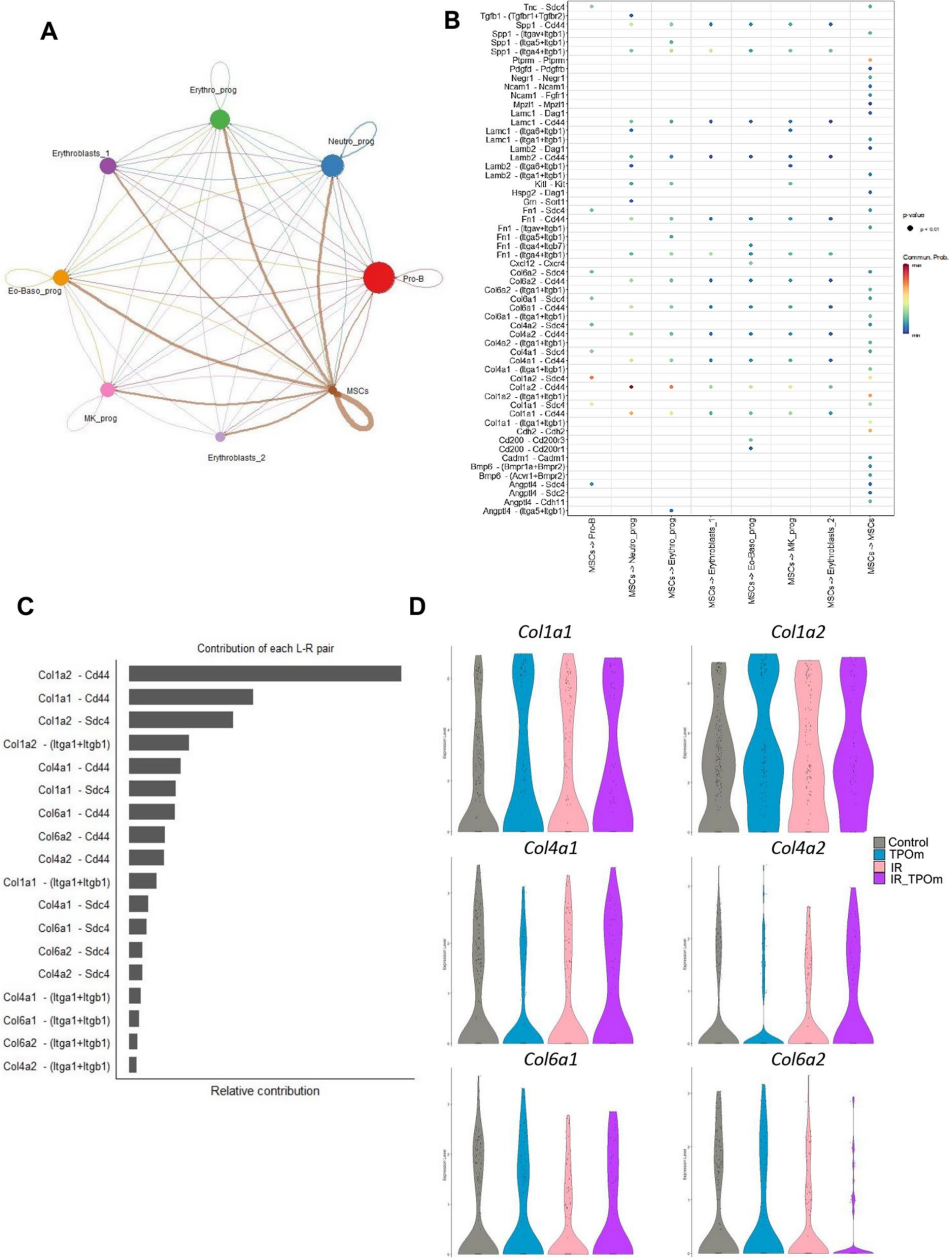

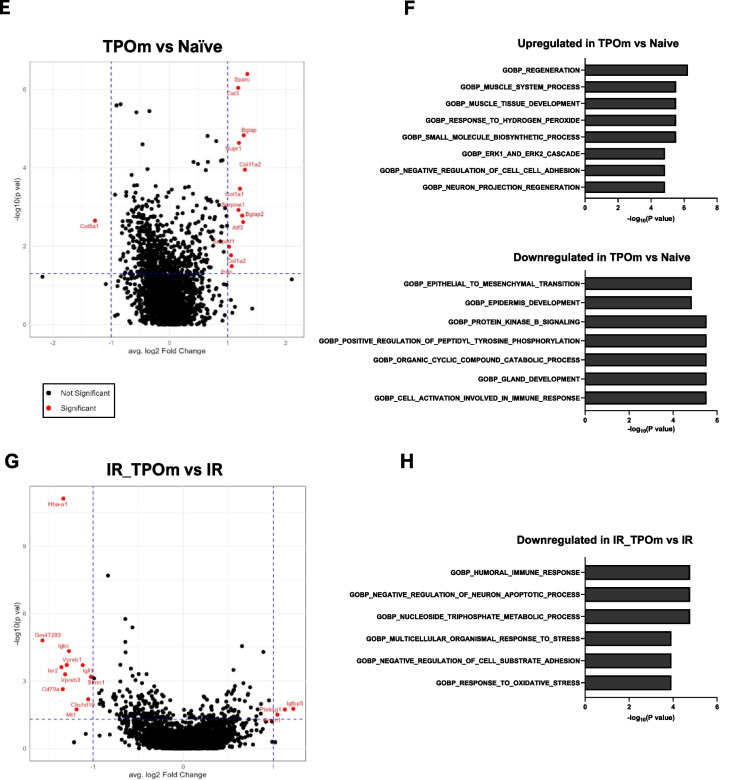
TPOm stimulates the interaction of MSCs with other cell clusters by upregulating the regeneration pathway and collagens expression. **A** Chord diagram of cell-to-cell communications between MSCs and other identified clusters of the TNC in the BM. **B** MSCs (sender) and other identified clusters (receivers) interaction by ligand and receptor. **C** Relative contributions of each ligand-receptor interaction. **D** Violin plots of the expression levels of different collagens expressed by MSCs iterated in each treatment group. **E** Volcano plot of differentially expressed genes of TPOm *vs.* Naïve groups and **F** Gene Ontology pathways significantly overrepresented among up- and down-regulated genes **G** Volcano plot of differentially expressed genes of irradiated (IR) *vs.* TPOm-irradiated (IR_TPOm) groups and **H** Gene Ontology pathways significantly overrepresented among down-regulated genes. Differential expression was performed with MAST model adjusting for Sex, nCount_RNA, percent.mt, S. Score

To explore cell–cell interaction among the different clusters, we used the CellChat [46] program to analyze the single-cell RNA sequencing data. The analysis revealed that MSCs acted as a central signaling hub, engaging in robust interactions with other clusters in the dataset (Fig. 6A). Identification of MSCs was based on the expression levels of several genes, including canonical MSC markers such as *Pdgfra* and *Lepr (*Additional file 1: Figure S6A). The predicted ligand-receptor communications from MSCs to each cluster, along with the intensity of these interactions is illustrated in Fig. 6B. Collagens expressed in MSCs emerged as the primary contributors to the cell–cell interactions, with *Col1a2-Cd44* exhibiting the highest contribution (Fig. 6C). *Cd44* was highly expressed in Neutro_prog, Mk_prog, and Erythro_prog (Fig. 6B and Additional file 1: Figure S6B). Another significant molecule in mediating cell–cell interactions was *Sdc4,* which was highly expressed in Pro-B cells and MSCs (Fig. 6B and Additional file 1: Figure S6B). We further analyzed the expression levels of several genes in the collagen family in each experimental group. In TPOm-treated mice, there was an increased expression of *Col1a1* and *Col1a2* in MSCs, and irradiation also heightened this expression (Fig. 6D). Particularly in the irradiated groups, TPOm treatment increased the expression of *Col1a2, Col4a1,* and *Col4a2* in MSCs compared to radiation alone, while it decreased the expression of *Col6a2* (Fig. 6D).

We further explored the expression of genes in MSCs that might be influenced by TPOm treatment. MSCs inherently expressed canonical EC ligands, such as VEGFs, which would contribute to EC regeneration (Additional file 1: Figure S6C). Differential expression analysis between TPOm treatment and naïve mice revealed that one gene, *Col8a1*, was significantly downregulated, and 12 genes were significantly upregulated, including various collagens and osteoblastic genes (Fig. 6E). The single cell pathway analysis (SCPA) using Gene Ontology Biological Pathways database (GOBP) of differentially expressed genes demonstrated a notable increase in the regeneration pathway in TPOm-treated mice compared to naïve (Fig. 6F). When comparing the differential expression between TBI and TBI plus TPOm-treated mice, 11 genes were significantly downregulated, and 3 genes were significantly upregulated (Fig. 6G). The SCPA of these genes indicated that the humoral immune response, multicellular organismal response to stress, and response to oxidative stress were significantly downregulated in the TPOm-treated mice after TBI (Fig. 6H). Together, these results suggest that TPOm upregulates several genes in the collagen family in MSCs, promoting their interaction with other hematopoietic TNCs in the BM. Moreover, TPOm stimulates regeneration and suppresses the humoral immune response in mice with treatment alone or after TBI, respectively.

## Discussion

In today’s geopolitical climate, individuals face the looming threat of exposure to high doses of ionizing radiation due to nuclear or radiological incidents, which carries the risk of developing ARS. In addition, patients undergoing myeloablative BM transplant conditioning suffer from radiation-induced toxicities and mortality. The hematopoietic system, consisting of highly proliferative stem cells, stands out as one of the most susceptible organ to radiation-induced injury [47]. In established animal models for TBI, four FDA-approved drugs targeting the hematopoietic system–Neupogen, Neulasta, Leukine, and Nplate (Romiplostim)-have demonstrated efficacy in increasing HSPCs in irradiated animals [2, 48–50]. However, their activity regulating the BM microenvironment for HSPC regeneration remains unexplored. In this study, we have evaluated the potential of TPOm (JNJ-26366821) as a radiation MCM [37] and agent than can mitigate radiation induced toxicities, focusing on its role in regulating BM vascular and stromal niches for HSPC regeneration in mice exposed to TBI from ^137^Cs and X-ray sources.

Our findings show that TPOm effectively expanded HSPCs, ECs, and MSCs in the BM of both healthy and irradiated mice. TPOm also significantly improved the 30-day survival of TBI-exposed mice, a necessary endpoint for evaluating drug efficacy in treating H-ARS according to the FDA animal rule [2]. Furthermore, our study reveals a novel activity of TPOm in alleviating BM vascular dilation in the sinusoidal niche and maintaining the arterioles of the arteriolar niche post-TBI. TPOm reduced vascular permeability, a typical consequence of exposure to high doses of radiation and increased the levels of VEGF-C in BM and serum. scRNA-seq analysis unveiled another novel function of TPOm in upregulating the expression of specific collagens in MSCs, thereby promoting their interaction with other rare hematopoietic progenitors in the BM. Additionally, TPOm upregulated regeneration and dampens the humoral response in MSCs.

We have verified that TPOm exhibited functionality akin to endogenous TPO by exerting its role as a regulator of platelet production from megakaryocytes through the differentiation of HSCs [29]. TPOm was developed by screening peptides capable of binding to c-MPL with a phage display library [51]. After a single *sc* injection, TPOm significantly increased the number of megakaryocytes in the BM of the healthy mice on days 3 and 6 which is in line with its role of promoting Mk differentiation. Moreover, TPOm induced a significant expansion of the HSPCs, consistent with prior studies demonstrating direct binding of TPO to HSCs and the expansion of LSK cells [52]. Recognizing the critical role of vascular and stromal niches in the BM for HSPC regeneration [9, 24, 44], our study examined the broader effects of TPOm treatment on other constituents of the BM microenvironment. We observed a significant increase in EPCs, ECs, AECs, SECs, and MSCs in the BM of healthy mice post-TPOm treatment. Notably, some organ-specific ECs expressing c-MPL, such as liver sinusoidal ECs and human umbilical vein ECs, have been reported [53, 54] suggesting the potential of direct interactions of ECs with TPOm. Furthermore, investigators have found that BM osteoblasts and -clasts express c-MPL [55]. Osteoblasts are derived from BM MSCs which may explain how TPOm is interacting with MSCs at homeostasis and after irradiation. While our findings point to a potential role of TPOm in the expansion of EC and MSC populations in the BM, the nature of this effect, whether direct or indirect, warrants further investigation.

For mice exposed to TBI at sublethal 7.0 Gy (^137^Cs) doses, the architecture and cellularity of BM were damaged, reflecting severe depletion of HSPCs, ECs, and EPCs that persisted for at least 14 days after TBI. With TPOm treatment, the architecture and cellularity of BM were more preserved, exhibiting less hemorrhage and adipocytes. A significant recovery of MPCs, LSK cells, and ST-HSCs by day 7 was observed in TBI mice treated with TPOm. Similarly, recent studies demonstrated that endogenous TPO, mainly produced from liver, promotes the regeneration of HSCs after chemo- and radio-induced myeloablation, an example of cross-organ signaling [56, 57]. Moreover, TPOm increased EPCs at day 7 after TBI, which would differentiate into mature ECs, resulting in a marked elevation of EC counts starting from day 10 after TBI.

Next, we evaluated the effectiveness of TPOm as a potential radiation MCM by subjecting mice to lethal doses of radiation: 8.8 Gy (^137^Cs), 6.7 Gy (X-ray), and 7.2 Gy (X-ray). Administering TPOm 24 h post-TBI resulted in a significant increase in 30-day survival rates at all three radiation doses, exceeding the vehicle-treated mice by at least 45%. Notably, TPOm demonstrated its efficacy not only in male mice but also in improving the survival of female mice exposed to X-ray irradiation. In addition to the enhanced survival rates, TPOm treatment effectively mitigated body weight loss following TBI. This mitigative effect aligns with our previous study, which demonstrated that TPOm significantly increased the survival of CD2F1 and C57BL/6J mice exposed to TBI from a ^60^Co γ-radiation source in a dose-dependent manner [37]. An important consideration is that various radiation sources, as described in our prior publication, can have significantly different effects on the composition of the BM depending on the dose; as such, γ-radiation and X-rays at isodoses are not equivalent [41]. In our current study, we administered a single dose of TPOm. Further studies are warranted to explore the pharmacodynamics of TPOm in mice to ascertain if additional doses could further improve survival rates post-TBI.

Our findings are consistent with the activity of other c-MPL agonists in mitigating H-ARS. For instance, administration of rhTPO enhanced the HSPC recovery in irradiated mice and significantly improved the survival of both mice and non-human primates exposed to lethal TBI [58]. Romiplostim, another TPO mimetic recently approved by the FDA to treat patients acutely exposed to myelosuppressive doses of radiation, has also demonstrated its efficacy in conferring a survival benefit in murine and non-human primate models of H-ARS [38, 59, 60]. To our knowledge, this is the first study to examine the role of TPOm in mitigating radiation-induced vascular and stromal injuries to support hematopoietic regeneration, marking a significant advancement in our understanding of TPO’s multifaceted mitigative mechanisms.

The vasculature within the BM can be divided into sinusoids and arterioles [17]. In mice exposed to TBI, we observed increased vessel dilation of the sinusoids, quantified by the area of VEGFR3, which was reduced by TPOm treatment. In the arteriolar niche, the structure of arterioles remained intact after lethal irradiation, albeit a marked decrease in the number of Sca-1^+^ cells was observed. Previous studies have reported differences in radiosensitivity between sinusoids and arterioles in the BM [9, 13]. The impact of TPOm on vasculature was further evident in the reduction of vascular dye leakage throughout the body of TBI mice, as detected by IVIS imaging. Likewise, we demonstrated that TPOm increased the levels of VEGFs in the BM and serum. Our findings are supported with previous reports indicating that TPO released from BM stromal cells can bind to HSCs to stimulate VEGF, implying a potential role in vascular regeneration [25, 61]. Vascular swelling after irradiation can be alleviated by HSC transplant supplemented with VEGF-A [9], and MSC-secreted VEGF-C has been shown to be crucial in regeneration of the vascular niche after irradiation [24]. Remarkably, TPOm distinctively increased VEGF-C levels within the BM and serum in healthy and irradiated mice. Consequently, TPOm may exert beneficial effects on regeneration and recovery of ECs post-irradiation, which subsequently affects BM HSPCs, potentially through the release of VEGFs.

To investigate the effect of irradiation with and without TPOm treatment on BM MSCs, we isolated the TNCs from the BM using cell sorting for single-cell RNA sequencing. Previous studies have established that the CD45^−^, TER-119^−^, CD31^−^ fraction of the BM is enriched with a heterogeneous population of MSCs and devoid of hematopoietic, erythroid, and endothelial cells [45]. However, recent findings have challenged this notion, revealing that TNCs contain cells of hematopoietic origins, particularly B lymphoid and erythroid lineages, which are dependent on signals from MSCs [23]. Our scRNA-seq data revealed a significant loss of Mk_prog, Neutro_prog, and Eo-Baso_prog populations after irradiation, with TPOm treatment mitigating this loss to some extent. Conversely, B lymphoid and erythroid lineages expanded after irradiation, with the erythroid clusters showing further enhancement with TPOm treatment. One study reveals that B lymphoid lineage and plasma cells derived from BM are resistant to radiation [62]. The elevation of Ter-119^low/−^ erythroid cells, a hallmark of stress-erythropoiesis, is typical after irradiation [63]. Further, TPO has been demonstrated to synergize with erythropoietin (EPO) and support erythroid recovery following myeloablative injury [64]. These findings suggest a potential role for TPOm in influencing the dynamics of various hematopoietic lineages post-irradiation.

The scRNA-seq data also uncovered the pivotal role of MSCs as central regulators of various hematopoietic TNCs. Interactions between MSCs and the other clusters were predominantly mediated by Col1a2/Col1a1 and syndecan-4 (Sdc4) or Cd44. Specifically, Sdc4 exhibited high expression on the Pro-B cluster and has been shown to modulate cell migration and adhesion [65]. Alternatively, the Neutro_prog, Erythro_prog, and Mk_prog clusters predominantly interacted with MSCs through Cd44, which is known for its critical functions in cell migration, adhesion, and homing [66]. Moreover, Cd44 has been used to differentiate stages of erythroid lineage development [67]. Sdc4 and Cd44 are known as cell-surface heparan sulfate proteoglycans that are indispensable for humoral immune system development and maintenance of hematopoiesis, in general [68]. These findings collectively suggest that MSCs play a crucial role in maintaining early B lymphoid and erythroid cells, priming them for HSPC recovery after irradiation. It is noteworthy that TPOm appears to augment these interactions, indicating a potential enhancement of MSC-mediated recovery of hematopoietic cells after irradiation.

TPOm has undergone thorough nonclinical toxicology evaluations, including chronic toxicity studies, and no issues have been identified that would preclude its clinical development [33]. A Phase 1 clinical study involving healthy volunteers further supported the safety and tolerability of TPOm. Particularly, TPOm dose-dependently elevated platelet counts and increased total colony-forming unit (CFU) counts compared to the placebo, with no evidence of antibody formation against endogenous TPO in humans [69].

**Fig. 7:**
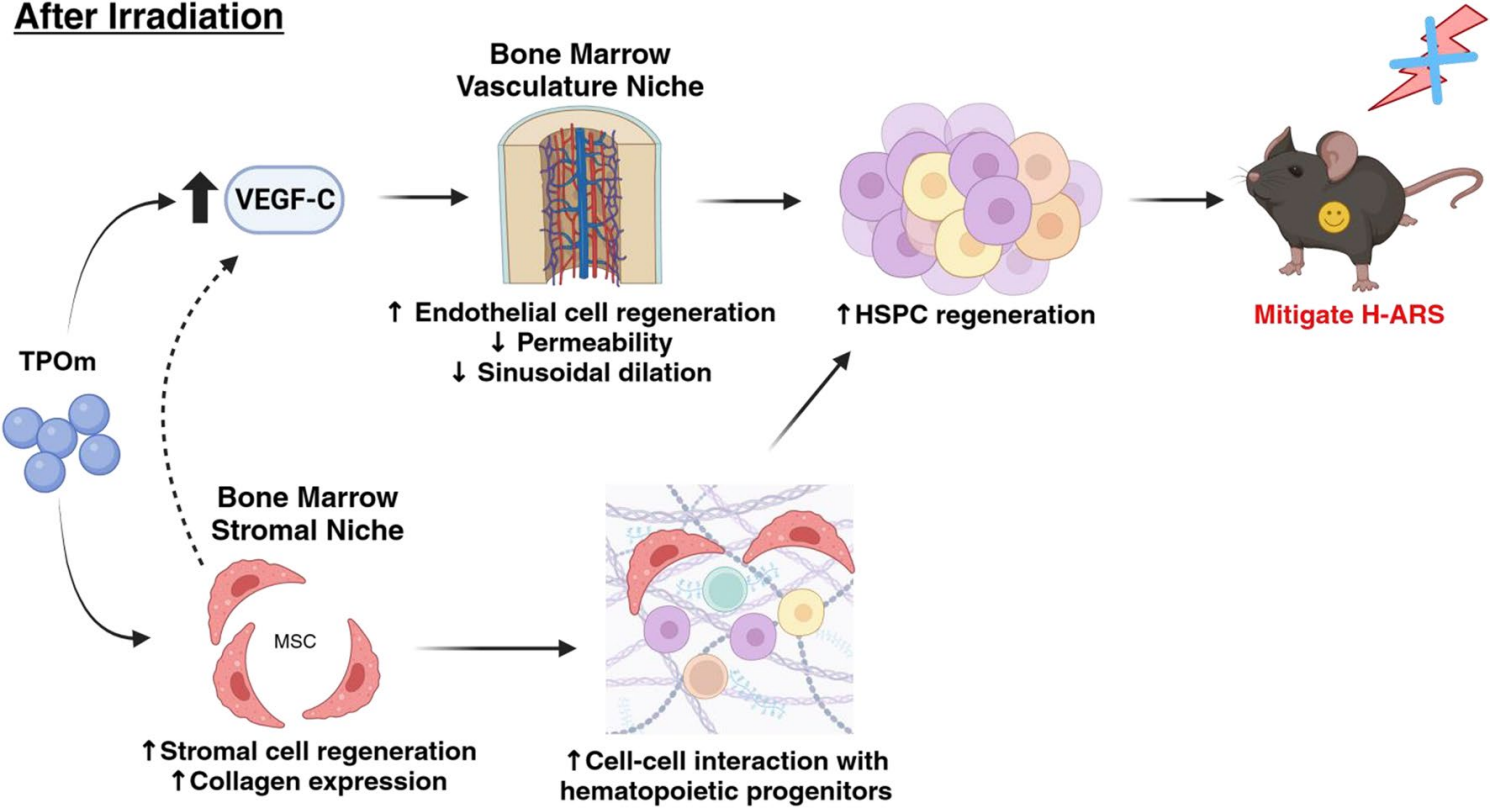
Summary of TPOm’s effect on BM vascular and stromal niches for HSPC regeneration after irradiation to mitigate H-ARS

In conclusion, our study has unveiled novel functions of TPOm (JNJ-26366821) in regulating the vascular and stromal niches in the BM, fostering the regeneration of HSPCs in irradiated mice. TPOm’s stimulation of VEGF secretion contributed to the maintenance vascular integrity in irradiated mice. Additionally, TPOm promoted MSCs to interact with other progenitors in the BM. These TPOm-induced effects collectively resulted in a significant improvement in the survival of the TBI mice, a model of H-ARS (Fig. 7). Taken together, TPOm is positioned as a clinical ready drug, meriting further development as radiation MCM for potential FDA approval.

## Materials and methods

### Key resources table

**Table:** 

Reagent or resource		Source	Identifier
Antibodies			
Hematopoietic stem and progenitor cell analysis (used 1:100 dilution unless otherwise noted)	PerCP-Cyanine5.5 anti-mouse Lineage	BD Biosciences	Cat#561317; RRID:AB_10612020
	FITC anti-mouse Ly-6A/E (Sca-1, clone D7)	Biolegend	Cat# 108106, RRID:AB_313343
	Alexa Fluor 700 anti-mouse Ly-6A/E (Sca-1, clone D7)	Biolegend	Cat# 108142, RRID:AB_2565959
	APC anti-mouse CD117 (c-kit, clone 2B8)	Biolegend	Cat# 105811, RRID:AB_313220
	PE/Cyanine7 anti-mouse CD41 (clone MWReg30; 1:200)	Biolegend	Cat# 133915, RRID:AB_11125173
	Alexa Fluor 700 anti-mouse CD34 (clone RAM34; 1:50)	BD Biosciences	Cat# 560518, RRID:AB_1727471
	Brilliant Violet 421 anti-mouse CD34 (clone SA376A4)	Biolegend	Cat# 152208, RRID:AB_2650766
	Pacific Blue anti-mouse CD150 (clone TC15-12F12.2)	Biolegend	Cat# 115924, RRID:AB_2270307
	Brilliant Violet 711 anti-mouse CD150 (clone TC15-12F12.2)	Biolegend	Cat# 115941, RRID:AB_2629660
	APC/Cyanine7 anti-mouse CD48 (clone HM48-1; 1:200)	Biolegend	Cat# 103431, RRID:AB_2561462
	Brilliant Ultra-Violet 395 anti-mouse CD48 (clone HM48-1; 1:200)	BD Biosciences	Cat# 740236, RRID:AB_2739984
Endothelial/ stromal cell analysis (used 1:100 dilution unless otherwise noted)	PerCP-Cyanine5.5 anti-mouse CD45 (clone 30-F11; 1:200)	Biolegend	Cat# 103132, RRID:AB_893340
	PerCP-Cyanine5.5 anti-mouse TER-119 (clone TER-119; 1:200)	Biolegend	Cat# 116228, RRID:AB_893636
	APC anti-mouse CD31 (clone 390)	Biolegend	Cat# 102409, RRID:AB_312904
	FITC anti-mouse CD31 (clone MEC13.3)	Biolegend	Cat# 102506, RRID:AB_312913
	Alexa Fluor 647 anti-mouse CD31 (clone MEC13.3)	Biolegend	Cat# 102516, RRID:AB_2161029
	Alexa Fluor 647 anti-mouse VE-Cadherin (clone BV13)	Biolegend	Cat# 138006, RRID:AB_10569114
	FITC anti-mouse CD62P (clone RB40.34)	BD Biosciences	Cat# 553744, RRID:AB_395026
	Brilliant Violet 605 anti-mouse CD140α (clone APA5)	Biolegend	Cat# 135916, RRID:AB_2721548
	PE anti-mouse CD51 (clone RMV-7)	Biolegend	Cat# 104106, RRID:AB_2129493
	Unconjugated anti-mouse VEGF Receptor 2 (clone D5B1)	Cell Signaling	Cat# 9698, RRID:AB_11178792
	Unconjugated anti-mouse VEGF Receptor 3	R&D	Cat# AF743, RRID:AB_355563
	Unconjugated anti-mouse Leptin Receptor	R&D	Cat# AF497, RRID:AB_2281270
	Biotinylated anti-mouse Leptin Receptor	R&D	Cat# BAF497, RRID:AB_2296953
	Biotinylated anti-mouse CD202b (TIE2, clone TEK4)	Biolegend	Cat# 124006, RRID:AB_2203221
	AffiniPure F (ab’)_2_ Fragment Donkey Anti-Goat IgG (H + L) Alexa Fluor 594	Jackson ImmunoResearch	Cat# 705-586-147, RRID:AB_2340434
	AffiniPure F (ab’)_2_ Fragment Donkey Anti-Rabbit IgG (H + L) Alexa Fluor 647	Jackson ImmunoResearch	Cat# 711-606-152, RRID:AB_2340625
Chemicals, Peptides, and Recombinant Proteins			
	Thrombopoiein mimetic (TPOm)	Janssen Pharmaceuticals	N/A
	Dispase II, powder	Gibco	Cat# 17105041
	Collagenase, Tyoe IV, powder	Gibco	Cat# 17104019
	Hank’s balanced salt solution (HBSS)	Gibco	Cat# 24020117
	Paraformaldehyde	Electron Microscopy Sciences	Cat# 15710
	10% Neutral buffered formalin	Fisher	Cat# F8775
	1X ACK lysing buffer	Lonza	Cat# 10-548E
	AngioSense750 EX	PerkinElmer	Cat# NEV10011EX
	Decal™ Decalcifier	StatLab	Cat# 1211-1
	Commercial Assays		
	Pierce™ BCA Protein Assay Kit	Thermo Scientific	Cat# 23225
	VEGF-A ELISA	R&D	Cat# MMV00, RRID:AB_2847842
	VEGF-C ELISA	Novus Biologicals	Cat# NBP2-78893, RRID: AB_3083672
	Chromium Next GEM Single Cell 3’ GEM, Library & Gel Bead Kit v3.1, 4 rxns	10X Genomics	Cat# 1000128
Deposited Data			
	Single-cell RNA-seq	This paper	PRJNA1086455
Experimental Models: Organisms/Strains			
	*Mus musculus*: C57BL/6 J	Jackson Laboratories	Stock #000664
	*Mus musculus*: B6.Cg-*Tg (Tek-cre)1Ywa*/J	Jackson Laboratories	Stock #008863, RRID:IMSR_JAX:008863, PMID: 11161575
	*Mus musculus*: B6.Cg-*Gt (ROSA)26Sor*^*tm14 (CAG−tdTomato)Hze*^/J	Jackson Laboratories	Stock #007914, RRID:IMSR_JAX:007914, PMID: 20023653
Software and Algorithms			
	GraphPad Prism	GraphPad	v10.1, RRID:SCR_002798
	FlowJo	BD Biosciences	v10.1, RRID:SCR_008520
	Living Image	Perkin Elmer	v4.3.1, RRID:SCR_014247
	Volocity	Quorum Technologies	v6.5.1, RRID:SCR_002668
	Slidebook	Intelligent Imaging Innovations	v6.0, RRID: SCR_014300
	FIJI	ImageJ	v1.53c, RRID:SCR_002285
	CaseViewer (SlideViewer)	3DHISTECH	v2.4.0.119028, RRID:SCR_024885
	CellRanger	10X Genomics	v7.0.1, RRID:SCR_017344
	R	http://www.r-project.org/	RRID:SCR_001905
	CellChat	http://www.cellchat.org/	v1.6.1, RRID:SCR_021946
	Seurat	https://satijalab.org/seurat/	v4.4.0, RRID:SCR_016341
	MAST	https://rglab.github.io/MAST/	v1.26.0, RRID:SCR_016340
	SCPA	https://jackbibby1.github.io/SCPA/	v1.5.4, RRID:SCR_024909

### Animals

C57BL/6J (wild-type; stock no. 000664), B6.Cg-*Tg (Tek-cre)1Ywa*/J (TIE2-cre; stock no. 008863), B6.Cg-*Gt (ROSA)26Sor*^*tm14 (CAG−tdTomato)Hze*^/J (tdTomato; stock no. 007914) mice were purchased from Jackson Laboratories (Bar Harbor, ME). B6.Cg-*Tg (Tek-cre)1Ywa*/J and B6.Cg-*Gt (ROSA)26Sor*^*tm14 (CAG−tdTomato)Hze*^/J were crossed together to generate constitutive Tek-cre; tdTomato (TIE2-tdTomato) mice in our facilities. All mice were acclimated for 1 week prior to experiments and group housed (no more than 5 per cage) in pathogen-free conditions under a 14:10 h light:dark cycle. Moreover, mice were housed at 20 °C to 22 °C with 30–70% humidity and fed ad libitum* (*Lab Diet 5001). To limit pathogen transmission, water was acidified to a pH of 2.5 to 3.0 with HCl for survival studies. All experiments were carried out using gender-matched littermate controls where appropriate. All mice in this study were used at 9–14 weeks of age. Both males and females were used for experiments.

### Preparation and injection of TPOm (JNJ-26366821)

TPOm was supplied as a powder for reconstitution at 1 mg/mL in sterile PBS. TPOm dosing formulations were stored protect from light, refrigerated (set to 2–8 °C) pending use for dosing within one day of preparation. Drug substance and stock solutions were stored protected from light in a − 80 °C freezer. Stock solutions in the concentration at 1 mg/mL can be stored in the above referenced freezer conditions for up to 8 weeks. Either TPOm or its vehicle were injected once subcutaneously at the nape, 24 h post-TBI. Based on a dose-escalation experiments of TPOm from a prior study, mice were dosed at 0.3 or 1.0 mg/kg [37].

### Irradiation

For ^137^Cs TBI, mice were anesthetized with 60:9 mg/kg ketamine:xylazine, which was equivalent to about 100 μL/mouse, and placed in single chambers of a round brass animal holder for the Shepherd Mark I irradiator. Brass container was placed on a rotating plate to expose them to uniform total body γ-irradiation according to the manufacturer’s specifications with a dose rate of about 1.90 Gy/min. TBI doses of 7.00 and 8.80/9.00 Gy were used for sublethal and lethal doses, respectively.

For X-ray total body irradiation, using a CIX-3 orthovoltage source (Xstrahl), unanesthetized mice were placed into a Plexiglas jig. The X-ray irradiator was operated at 300 kVp, 10 mA with either 1 mm Cu at a dose rate of 1.89 Gy/min or 4 mm Cu filtration (for scRNA-seq data) at a dose rate of 1.12 Gy/min at a 40 cm source surface distance. All irradiation was performed in the morning. Doses and dosimetry were determined as described in our previous publication comparing ^137^Cs γ-radiation to orthovoltage X-rays [41].

### Complete blood count

At the time of euthanasia, mice were subjected to isoflurane overdose and blood was collected via cardiac puncture into K**2**EDTA coated microtainer tubes (BD Pharmingen, cat# 365967). Automated complete blood count with differential was performed using a Hemavet 950FS instrument (Drew Scientific).

### Bone marrow histology

Sternums were fixed in 10% neutral-buffered formalin (Fisher, cat# F8775) overnight at room temperature, followed by decalcification (StatLab, cat# 1211-1) overnight at room temperature. Sternums were then embedded in paraffin. Paraffin sections were cut at 5 µm intervals and stained with hematoxylin and eosin (H&E). Slides were imaged on the P250 Slide Scanner (3DHISTEC) using the 20 × objective. Megakaryocytes were quantified manually using FIJI (ImageJ v1.53c). Adipocytes were quantified using QuPath [70] software with the MarrowQuant script as described previously [40].

### Flow cytometry and cell sorting

For analysis of hematopoietic cells, femurs were flushed with 2% FBS-PBS 2 mM EDTA (FPE) buffer with a 21G needle and for analysis of endothelial and stromal cells, tibias were flushed and digested with 1 mg/mL of collagenase IV (Gibco, cat# 17104019) and 2 mg/mL of dispase (Gibco, cat# 17105041) in Hank’s balanced salt solution (HBSS) (Gibco, cat# 24020117) for a total of 30 min at 37 °C with an inversion at 15 min as previously described [12, 15]. After either procedure, cell pellet was resuspended in 1X ACK lysing buffer (Lonza, cat# 10-548E) to red blood cell lysis. The suspension was filtered through 70 µm nylon mesh and counted using TC20 automated cell counter (BioRad) in 0.4% (w/v) Trypan Blue.

For FACS analysis, cells were incubated with Live/Dead Zombie NIR fixable dye (Biolegend, cat# 423106) in PBS/2 mM EDTA at room temperature for 15 min. After, primary antibodies were diluted in FPE buffer and incubated with cells for 30 min at 4 °C. The antibodies used for hematopoietic stem and progenitor cell analysis were diluted at 1:100 unless otherwise specified (see Key Resources Table): PerCP-Cyanine5.5 anti-mouse Lineage Cocktail, FITC anti-mouse Ly-6A/E (Sca-1), Alexa Fluor 700 anti-mouse Sca-1, APC anti-mouse CD117 (c-kit), PE/Cyanine7 anti-mouse CD41 (1:200 dilution), Alexa Fluor 700 anti-mouse CD34 (1:50 dilution), Brilliant Violet 421 anti-mouse CD34, Pacific Blue anti-mouse CD150, Brilliant Violet 711 anti-mouse CD150, APC/Cyanine7 anti-mouse CD48 (1:200 dilution), Brilliant Ultra-Violet 395 anti-mouse CD48 (1:200 dilution). The antibodies used for endothelial/stromal cell analysis were diluted at 1:100 unless otherwise specified (see Key Resources Table): PerCP-Cyanine5.5 anti-mouse CD45 (1:200 dilution), PerCP-Cyanine5.5 anti-mouse TER-119 (1:200 dilution), APC anti-mouse CD31, FITC anti-mouse CD31, FITC anti-mouse CD62P, Brilliant Violet 605 anti-mouse CD140α, PE anti-mouse CD51, anti-mouse VEGF Receptor 2, AffiniPure F (ab’)_2_ Fragment Donkey Anti-Rabbit IgG (H + L) Alexa Fluor 647 (1:300 dilution), anti-mouse Leptin Receptor, AffiniPure F (ab’)_2_ Fragment Donkey Anti-Goat IgG (H + L) Alexa Fluor 594 (1:300 dilution), Streptavidin Alexa Fluor 488 (1:300 dilution), anti-mouse TIE2, Streptavidin PE-Cy5 (1:300 dilution). Cells were washed with 2% FBS-PBS solution and incubated with secondary antibodies for 30 min, if necessary. Cells were resuspended in FPE buffer and acquired on Cytek Aurora with SpectroFlo software or BD LSRII with FACS Diva software on flow cytometer. Cell sorting was performed on FACSAria Cell Sorter (BD Biosciences) as previously described [23]. Dead cells and debris were excluded by FSC, SSC, and Live/Dead staining. Data analysis was done through FlowJo (Tree Star, v10.1) software.

### Immunofluorescence imaging and analysis

In vivo staining of bone marrow endothelial cells was done via retroorbital perfusion of EC-specific antibodies Alexa Fluor 647 anti-mouse VE-Cadherin (5 μg, Biolegend, cat# 138006, clone BV13) and Alexa Fluor 647 anti-mouse CD31 (5 μg, Biolegend, cat# 102516, clone MEC13.3) for 15 min. Femoral bones were extracted and fixed overnight in 4% paraformaldehyde (PFA, Electron Microscopy Sciences, cat# 15710), incubated in 30% sucrose for at least 24 h and embedded in optical cutting temperature compound (OCT) (Fisher, cat# 4585). For whole-mount staining, bones were shaved on a cryostat until the bone marrow cavity was fully exposed. Bones were carefully harvested from melting OCT and stained in Eppendorf tubes with anti-mouse VEGFR3 (unconjugated, 5 μg/femur, R&D, cat# AF743) and Hoechst 33342 for nuclei staining (1:2000 dilution, Thermo Scientific, cat# 62249) as previously described [22].

Similarly, for whole-mount preparation of sternum, sternums were collected and bisected sagittally for exposure of the bone marrow cavity then fixed in 4% PFA for 30 min, washed 3X with PBS, then stained with FITC anti-mouse Ly-6A/E [Sca-1; (1:100 dilution, Biolegend, cat# 108106, clone D7)] and Hoechst 33342 for nuclei staining as previously described [7]. Images were acquired using a water immersion lens on the ZEISS AXIO examiner D1 microscope (Zeiss) with a confocal scanner unit, CSUX1CU (Yokogawa), and reconstructed in three dimensions with Slide Book software (Intelligent Imaging Innovations, v6.0) or analyzed using Volocity software (Quorum Technologies, v6.5.1). Briefly, original images were loaded into Volocity as.TIFF file formats. Brightness-contrast and noise reduction modifications were applied to each channel for the whole image. Quantification of vessel area and quantification of Sca-1^+^ cells were performed in Volocity.

### ELISA

Plasma was collected after complete blood count analysis using 8000 × *g* for 5 min to spin down whole blood and stored at − 80 °C. Bone marrow supernatant was collected by flushing a 200 μL of 1X PBS through two femurs. The solution was centrifuged at 300x*g* for 5 min to separate cells and supernatant was separately stored at − 80 °C. Protein concentrations for the BM supernatant was determined using BCA Protein Assay kit (Thermo Scientific, cat# 23,225) according to manufacturer’s instructions with BSA as a standard. ELISA for VEGF-A (R&D, cat# MMV00) and VEGF-C (Novus Biologicals, cat# NBP2-78893) were performed according to manufacturer’s instructions. Standard dilutions for plasma and 10 μg of total protein for BM supernatant were loaded for either ELISA for standardization.

### In vivo imaging

IVIS was performed on the Caliper Life Sciences IVIS Spectrum system. Mice were intravenously perfused with AngioSense750 EX (PerkinElmer, cat# NEV10011EX) on day 3 after irradiation as per manufacturer’s instructions. On day 5 after irradiation, mice were anesthetized with isoflurane (2% v/v with oxygen as the carrier gas) in an inhalation chamber (VetEquipt, cat# 911103) and maintained as mice were in the IVIS. The radiant efficiency, a relative measure of photon emission from the animal (photons/s/cm^2^), was measured in a standardized region of interest (ROI) with the variables of exposure time, binning, and focal length/stop also standardized. Fluorescence measurements were acquired with Living Image (Perkin Elmer, v4.3.1) and are expressed as a pseudocolor on a gray background, with red representing the lowest intensity and blue the highest.

### Library preparation and sequencing

Single-cell RNA sequencing libraries involved sorted bone marrow cells stained with CD45, TER-119, and CD31 markers. These libraries were generated from a total of ~ 20,000 individual cells, combining cell-multiplexing oligos (CMOs) from one male and one female mouse, contributing about ~ 10,000 cells each. The process involved generating cDNA within individual cell-gel bead emulsion micro-reactors, during which barcodes were added at both cellular and molecular levels. This barcoding allowed for the combination of the cDNA from individual cells for further library processing. Unique molecular barcodes (UMIs) were utilized to ensure that amplification artifacts did not distort the analysis. The prepared libraries underwent sequencing for 4000 M reads (PE150), with approximately 400 million read pairs for gene expression libraries and about 100 million read pairs for CMO libraries, all sequenced on an Illumina HiSeq 2500 system.

### Single-cell RNA sequencing analysis

Data were analyzed with a high-throughput next-gen sequencing pipeline. CellRanger (7.0.1) was used for data preprocessing. Seurat package (4.4.0) was harnessed for data analysis. Cells filtering was performed with following thresholds: nFeature_RNA > 200 & nFeature_RNA < 6000 & percent.mt < 5. Cells were identified based on their Seurat clustering and their positive markers as well as using specific markers expressions available from literature. Cell communications scores were calculated and visualized using CellChat package (1.6.1). Differential expression was calculated using MAST package (1.26.0) that allowed for adjustment to confounding variables (Sex, nCount_RNA, percent.mt, S.Score). Pathway analysis was performed with SCPA package (1.5.4) with MSigDB Mus musculus C5.BP pathway library.

### Statistical analysis

Statistical analysis and graphs were conducted and generated through GraphPad Prism (v10.1). Specific statistical details for each figure can be found at the end of each figure legend. Survival was analyzed using Kaplan–Meier curves with log-rank Mantel-Cox test. One-way ANOVA was used to compare three or more groups with a single control group using post hoc Dunnett test for multiple comparisons correction. Multiple Student’s *t* tests were performed for statistical analysis between vehicle and TPOm-treated with post hoc Holm-Sidak test for multiple comparisons. Outliers were determined using ROUT with a Q = 0.2% and excluded only in irradiated flow cytometry experiments (Fig. 2 and Additional file 1: Fig. S2). Naïve controls were excluded from statistical analysis and only shown as reference. No statistical method was used to determine sample size. All mice from experiments were randomized for each experimental group and investigators were not blinded to their allocation. n represent the number of mice used in each experiment which was replicated 2–4 times. Results were considered statistically significant when *p* < 0.05 and *p < 0.05, **p < 0.01, ***p < 0.001, ****p < 0.0001. All data are shown as mean ± SEM.

### Supplementary Information

**Additional file 1:** **Figure S1. **TPOm augments the frequency of hematopoietic stem and progenitor, endothelial, and stromal cells in murine bone marrow at homeostasis. (**A**) Gating strategy and representative flow plots for identifying various hematopoietic stem cell populations for naive and day 3 after TPOm treatment groups. (**B**) The frequency of live MPC, MkP, LSK, ST-, and LT-HSC of naïve and TPOm-treated mice (n=3-4/group) over time. (**C**) Gating strategy and representative flow plots for identifying various endothelial cell and stromal populations for naive and day 3 after TPOm treatment groups. (**D**) The frequency of live EC, EPC, AEC, SEC, and MSC of naïve and TPOm-treated mice (n=3-4/group) over time. Data are expressed as mean ± SEM. *p < 0.05 *vs.* naïve assessed by one-way ANOVA with post hoc Dunnett test for multiple comparisons. **Figure S2. **TPOm increases the frequency of hematopoietic stem and progenitor, endothelial, and stromal cells in murine bone marrow following 7.0 Gy sublethal total body irradiation. (**A**) The frequency of live MPC, MkP, LSK, ST-, and LT-HSC of naïve and TPOm-treated mice (n=4-29/group) over time. (**B**) The frequency of live EC, EPC, AEC, SEC, and MSC of naïve and TPOm-treated mice (n=4-29/group) over time. Data are expressed as mean ± SEM. *p < 0.05, **p < 0.01, ***p < 0.001 vehicle *vs*. TPOm-treated assessed by unpaired Student’s *t*-test with post hoc Holm-Sidak method for multiple comparisons. Outliers were determined using ROUT with a Q = 0.2%. **Figure S3. **TPOm increases survival of female mice and reduces hemorrhaging in the bone marrow of male mice after lethal total body irradiation.(**A**) Kaplan-Meier survival curve of vehicle and TPOm treated female mice for 30 days after 7.2 Gy X-ray TBI. (**B**) The percentage of body weight change over 30 days after 7.2 Gy X-ray TBI in female mice. (**C**) Representative H&E images of sternal bone marrow from 8.8 Gy (^137^Cs) irradiated vehicle and TPOm-treated mice on days 7 and 14 after irradiation. For survival the Log-rank (Mantel-Cox) test was used for curve comparison. Light blue arrow, adipocytes; dark blue box, hemorrhaging. Scale bar is 100 μm, For the percent weight change data are expressed as mean ± SEM. **Figure S4. **TPOm promotes secretion of VEGF-A and VEGF-C in healthy mice. (**A**, **B**) ELISA of VEGF-A in (**A**) serum and in (**B**) BM after TPOm treatment. (**C**, **D) **ELISA of VEGF-C in (**C**) serum and in (**D**) BM after TPOm treatment. Data are expressed as mean ± SEM. *p < 0.05 *vs.* naïve assessed by one-way ANOVA with post hoc Dunnett test for multiple comparisons. **Figure S5. **TPOm increases ***Mki67*** expression on megakaryocyte progenitors. (**A**) Cell cycle status of individual UMAP per treatment group. (**B**) Violin plots of the expression level of *Mki67* on megakaryocyte progenitors for each treatment group. **Figure S6. **MSCs express various collagens and VEGFs. (**A**) Dot plot of genes highly specific to the MSC cluster of the TNC in the BM. (**B**) Expression levels of different collagens and their interacting partners on each cluster. (**C**) Expression levels of different vascular growth factors secreted by each cluster.

## Data Availability

The datasets supporting the conclusions of this article are available in the Figshare repository, with the following link: https://figshare.com/s/79412a5256d7cd59b306. Single-cell RNA sequencing data are deposited under BioProject number PRJNA1086455. This paper does not report original code. All code used in this publication are publicly available online. Further information and requests for resources and reagents should be directed to and will be fulfilled upon reasonable request by the corresponding author, Dr. Chandan Guha (cguhamd@gmail.com).

## Abbreviations

TBI: Total body irradiation
BM: Bone marrow
H-ARS: Hematopoietic acute radiation syndrome
TPOm: Thrombopoietin mimetic, JNJ-26366821
HSPC: Hematopoietic stem and progenitor cell
MSC: Mesenchymal stromal cell
MCM: Medical countermeasure
LSK: Lineage^−^, Sca-1^+^, c-kit^+^
LT-HSC: Long-term hematopoietic stem cell
ST-HSC: Short-term hematopoietic stem cell
MPC: Myeloid progenitor cell
EPC: Endothelial progenitor cell
EC: Endothelial cell
AEC: Arteriolar endothelial cell
SEC: Sinusoidal endothelial cell
SCF: Stem cell factor
TNC: Triple negative cell; CD45^−^, TER-119^−^, CD31^−^
VEGF: Vascular endothelial growth factor
TPO: Thrombopoietin
rhTPO: Recombinant human thrombopoietin
PLT: Platelets
WBC: White blood cells
NE: Neutrophils
LY: Lymphocytes
MkP: Megakaryocyte progenitor
sc: Subcutaneous
LD_%DEATH/TIME_: Lethal dose
VEGFR: Vascular endothelial growth factor receptor
MK_Prog: Megakaryocyte progenitor in single-cell data
Neutro_Prog: Neutrophil progenitor in single-cell data
Eo-Baso_Prog: Eosinophil-Basophil progenitor in single-cell data
Erythro_Prog: Erythroid progenitor in single-cell data
SCPA: Single cell pathway analysis
GOBP: Gene ontology biological pathways
