# Supplementary figures and images for: Thrombopoietin mimetic stimulates bone marrow vascular and stromal niches to mitigate acute radiation syndrome
Source: Stem Cell Res Ther. 2024 Apr 29;15:123. doi: 10.1186/s13287-024-03734-z (PMC11057170; doi:10.1186/s13287-024-03734-z)

**Figure 1 supplement**

**A**

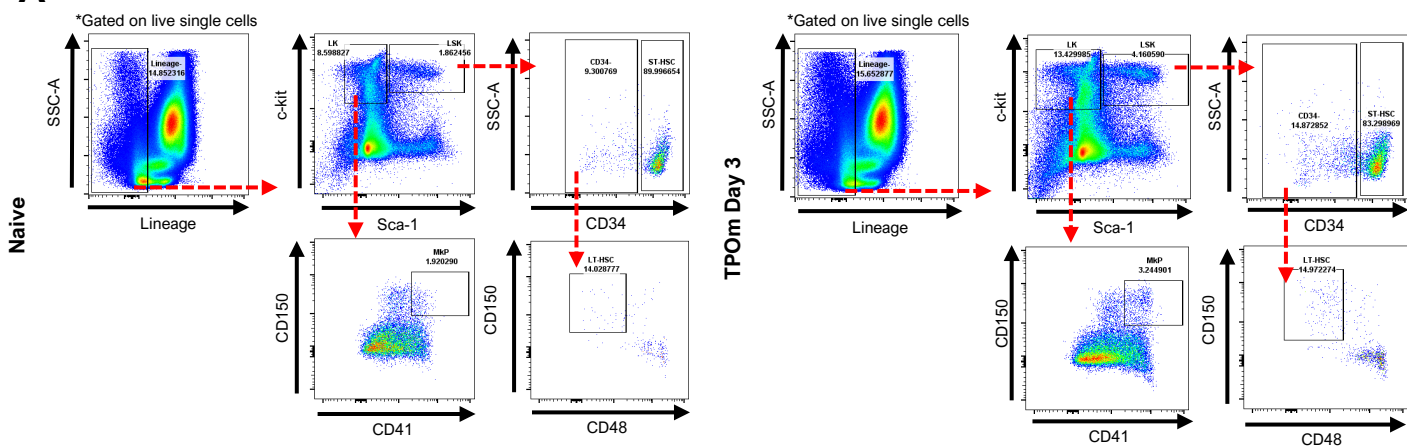

**B**

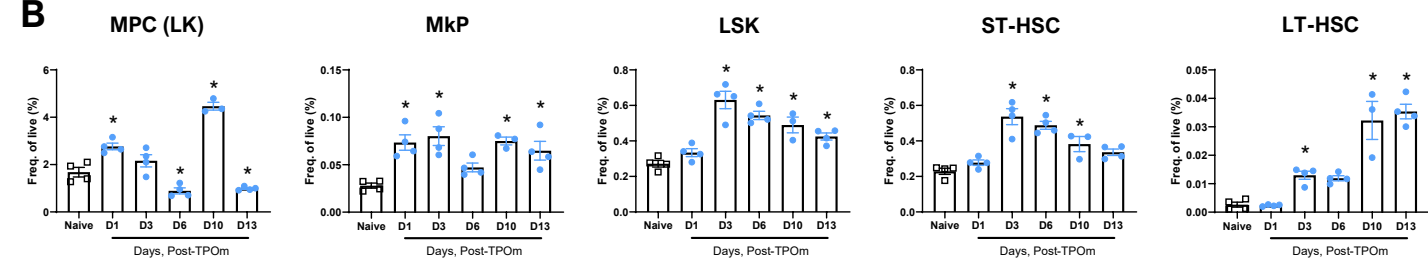

**C**

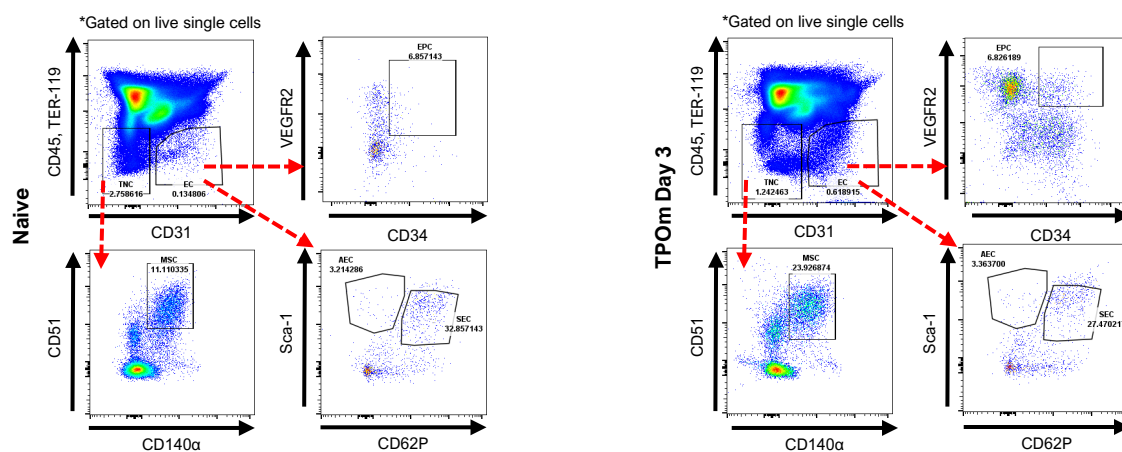

**D**

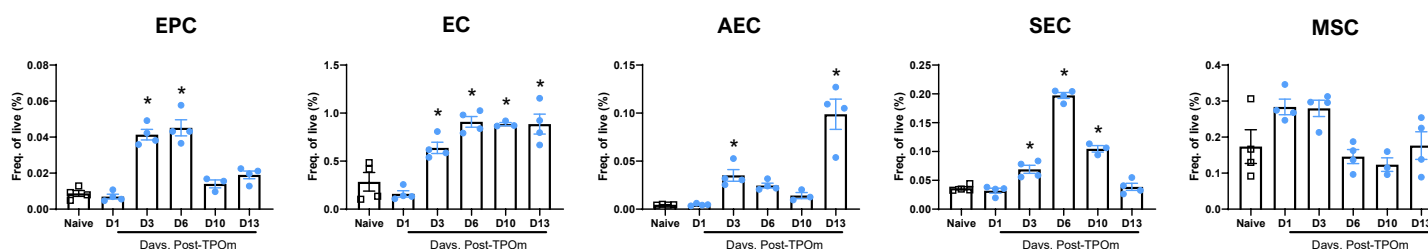

**A**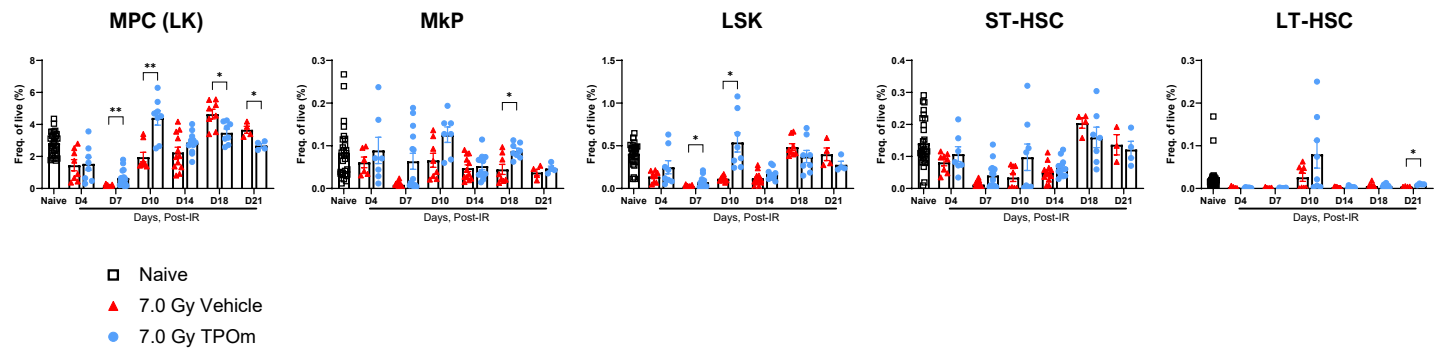**B**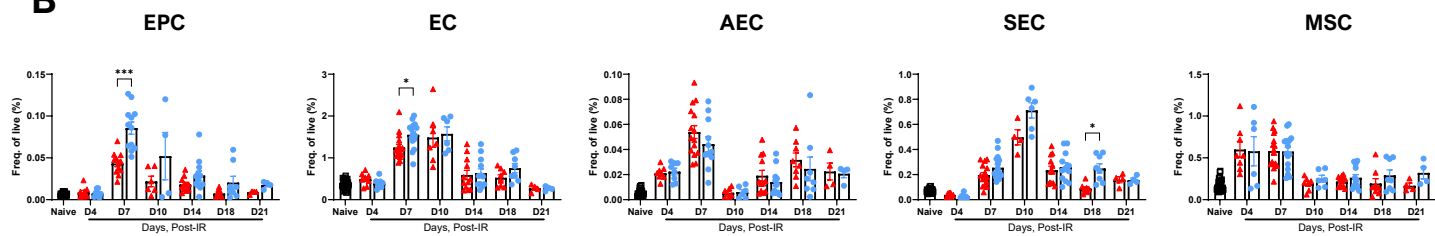

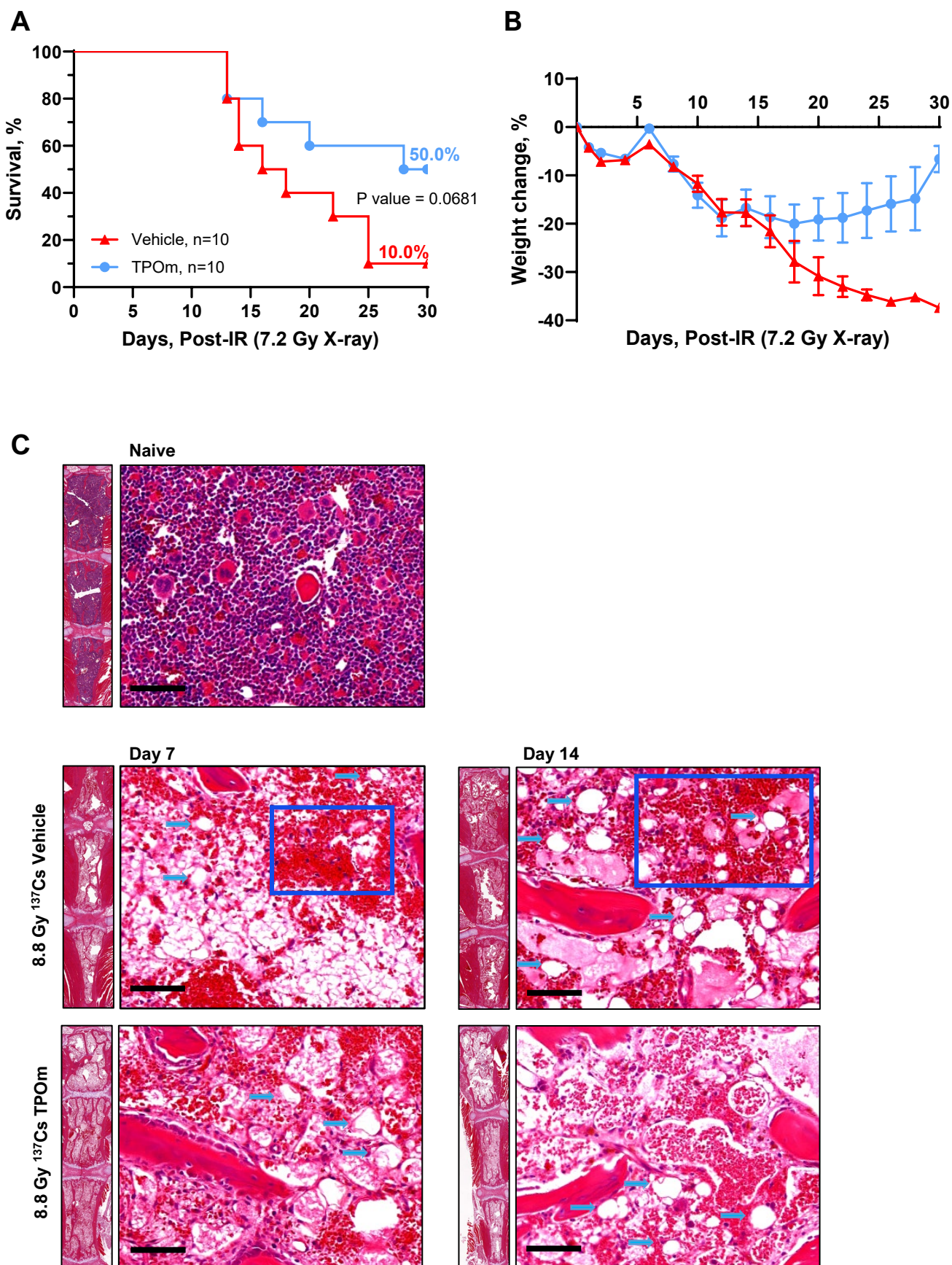

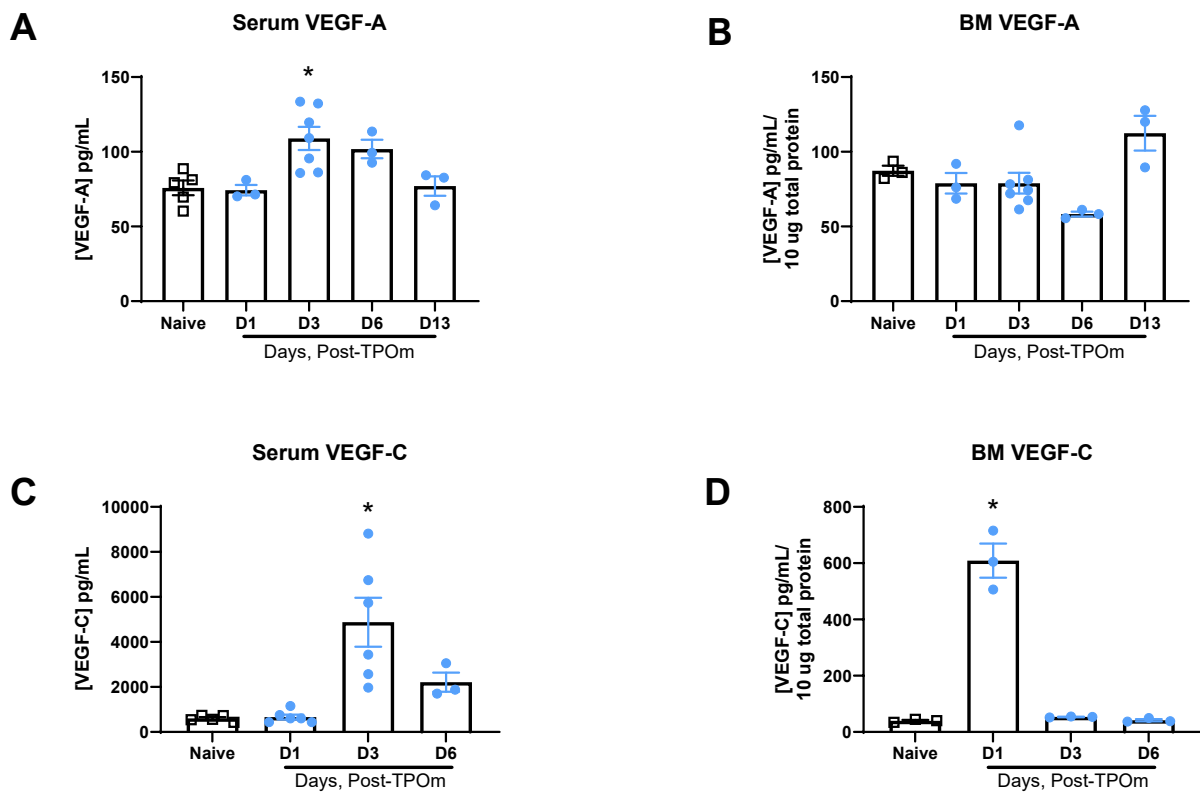

**A****MK\_Prog *Mki67***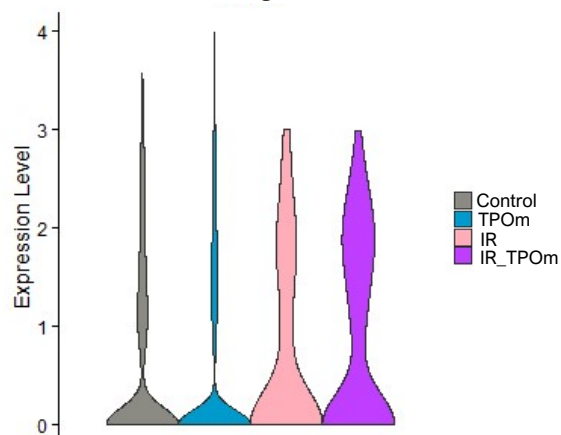**B****Control****TPOm****IR****IR\_TPOm**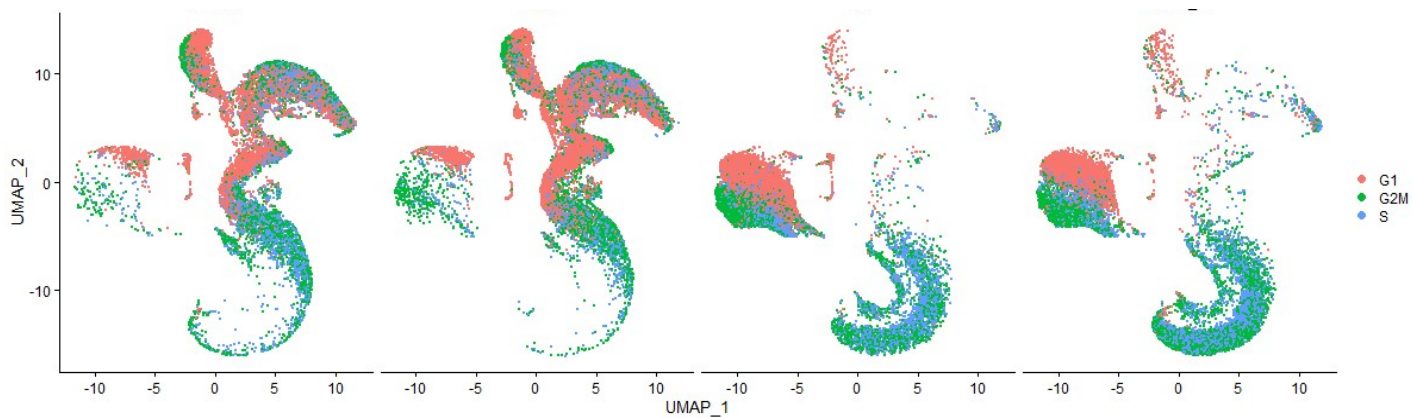

Figure 6 supplement

**A**

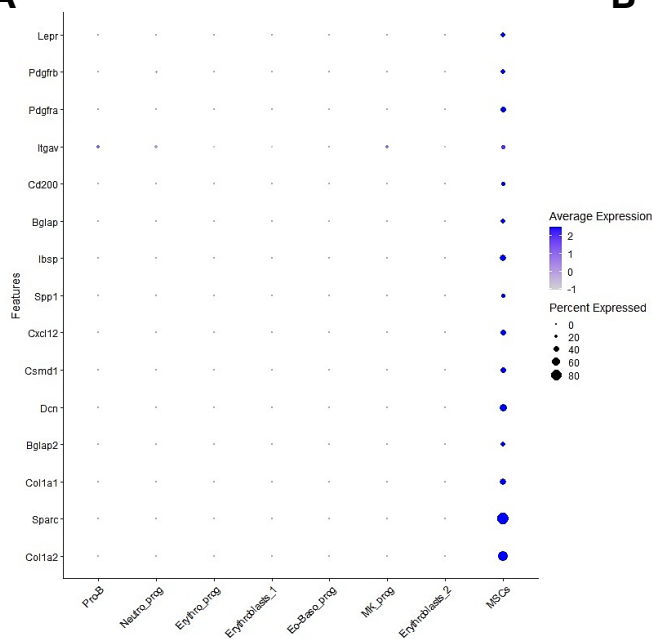

**B**

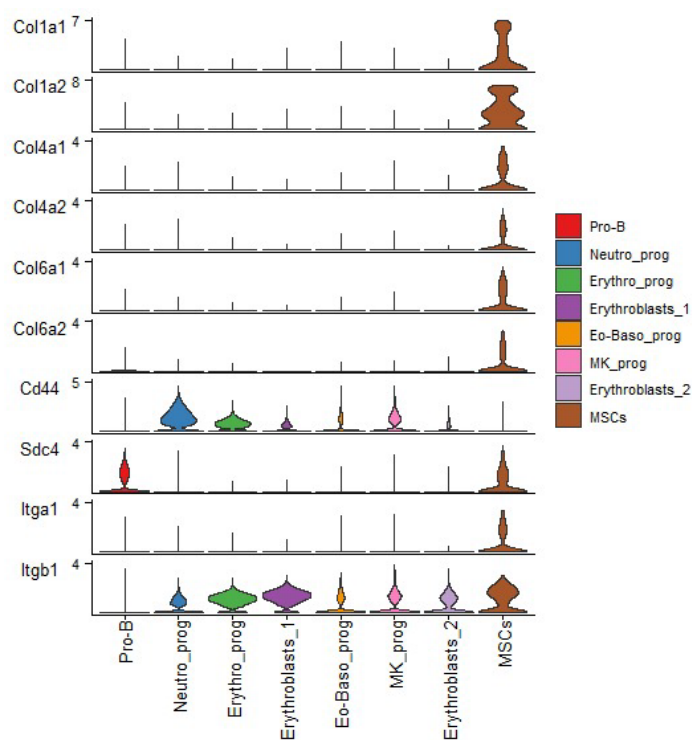

**C**

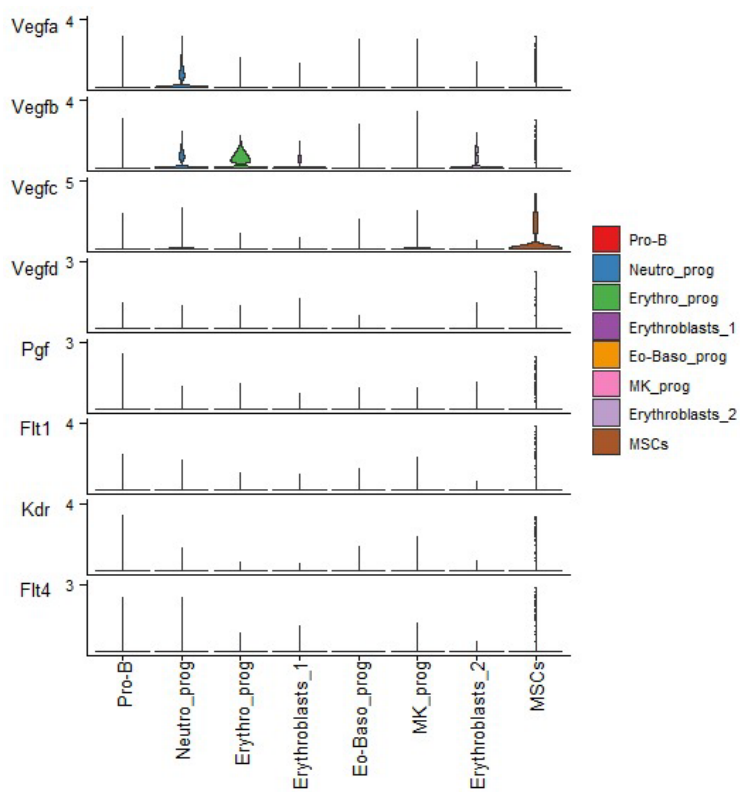

Supplement: Supplementary file 1 — Additional file 1: Figure S1. TPOm augments the frequency of hematopoietic stem and progenitor, endothelial, and stromal cells in murine bone marrow at homeostasis. (A) Gating strategy and representative flow plots for identifying various hematopoietic stem cell populations for naive and day 3 after TPOm treatment groups. (B) The frequency of live MPC, MkP, LSK, ST-, and LT-HSC of naïve and TPOm-treated mice (n=3-4/group) over time. (C) Gating strategy and representative flow plots for identifying various endothelial cell and stromal populations for naive and day 3 after TPOm treatment groups. (D) The frequency of live EC, EPC, AEC, SEC, and MSC of naïve and TPOm-treated mice (n=3-4/group) over time. Data are expressed as mean ± SEM. *p < 0.05 vs. naïve assessed by one-way ANOVA with post hoc Dunnett test for multiple comparisons. Figure S2. TPOm increases the frequency of hematopoietic stem and progenitor, endothelial, and stromal cells in murine bone marrow following 7.0 Gy sublethal total body irradiation. (A) The frequency of live MPC, MkP, LSK, ST-, and LT-HSC of naïve and TPOm-treated mice (n=4-29/group) over time. (B) The frequency of live EC, EPC, AEC, SEC, and MSC of naïve and TPOm-treated mice (n=4-29/group) over time. Data are expressed as mean ± SEM. *p < 0.05, **p < 0.01, ***p < 0.001 vehicle vs. TPOm-treated assessed by unpaired Student’s t-test with post hoc Holm-Sidak method for multiple comparisons. Outliers were determined using ROUT with a Q = 0.2%. Figure S3. TPOm increases survival of female mice and reduces hemorrhaging in the bone marrow of male mice after lethal total body irradiation.(A) Kaplan-Meier survival curve of vehicle and TPOm treated female mice for 30 days after 7.2 Gy X-ray TBI. (B) The percentage of body weight change over 30 days after 7.2 Gy X-ray TBI in female mice. (C) Representative H&E images of sternal bone marrow from 8.8 Gy (137Cs) irradiated vehicle and TPOm-treated mice on days 7 and 14 after irradiation. For survival [file 13287_2024_3734_MOESM1_ESM.pdf]
